# High‐Throughput Design of Magnetocaloric Materials for Energy Applications: MM´X alloys

**DOI:** 10.1002/advs.202206772

**Published:** 2023-04-20

**Authors:** Nuno M. Fortunato, Andreas Taubel, Alberto Marmodoro, Lukas Pfeuffer, Ingo Ophale, Hebert Ebert, Oliver Gutfleisch, Hongbin Zhang

**Affiliations:** ^1^ Institute of Materials Science TU Darmstadt Otto‐Berndt‐Str. 3 64287 Darmstadt Germany; ^2^ Institute of Materials Science Functional Materials TU Darmstadt Alarich‐Weiss‐Str. 16 64287 Darmstadt Germany; ^3^ Institute of Physics (FZU) of the Czech Academy of Sciences Cukrovarnická 10 Praha 16253 Czech Republic; ^4^ Department Chemie Universität München Butenandstr. 5‐13 81377 München Germany

**Keywords:** ab initio calculations, energy materials, high‐throughput screening, magnetocaloric effect

## Abstract

Magnetic refrigeration offers an energy efficient and environmental friendly alternative to conventional vapor‐cooling. However, its adoption depends on materials with tailored magnetic and structural properties. Here a high‐throughput computational workflow for the design of magnetocaloric materials is introduced. Density functional theory calculations are used to screen potential candidates in the family of MM'X (M/M’ = metal, X = main group element) compounds. Out of 274 stable compositions, 46 magnetic compounds are found to stabilize in both an austenite and martensite phase. Following the concept of Curie temperature window, nine compounds are identified as potential candidates with structural transitions, by evaluating and comparing the structural phase transition and magnetic ordering temperatures. Additionally, the use of doping to tailor magnetostructural coupling for both known and newly predicted MM'X compounds is predicted and isostructural substitution as a general approach to engineer magnetocaloric materials is suggested.

## Graphical abstract

Magnetic cooling requires optimized compounds, such as when magnetic and structural transitions to occur concurrently. A computational workflow for the discovery of magnetocaloric materials is proposed and implemented for a family of intermetallic compounds. Out of 274 stable compounds, nine exhibit are predicted to show structural transitions. Chemical disorder strategies are proposed for further improvement.

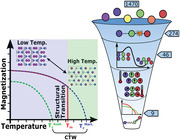

## 1 Introduction

Driven by climate change and worldwide economic development, the demand for cooling applications is increasing rapidly throughout the 21st century, resulting in an expected significant increase in energy consumption, related CO_2_ emissions and more widespread utilization of refrigerants with high global warming potential (GWP).^[^ ^1^ ^]^ Magnetic refrigeration exploits materials with a large magnetocaloric effect (MCE), through the application and removal of a magnetic field. This technology offers several advantages in comparison to the conventional vapor‐compression cycle, such as a lack of gases harmful to the environment (GWPs) and a higher energy efficiency.^[^ ^2^ ^]^ Therefore, there is a strong impetus to design and optimize magnetocaloric materials so that systems with optimal performance can be engineered for scaled‐up applications. From the physics point of view, one can differentiate between MCE systems with a second order magnetic transition and those with a first‐order transition, affecting the two prime figures of merit: the entropy changes and the adiabatic temperature change. Second‐order transitions are exemplified by Gd and related rare‐earth (RE) such as Gd‐Tb.^[^ ^3^ ^]^ However, commercial adoption is hindered due to the criticality of REs and by the environmental cost of material extraction and processing.^[^ ^4^ ^]^ In first‐order transitions, a high MCE is realized by a large and discontinuous magnetization change, e.g., by going from ferromagnetic (FM) to paramagnetic (PM) states, with the apparent advantage that a large MCE can be induced by smaller applied magnetic fields. This opens up a broader range of material families, such as La‐Fe‐Si and Heusler alloys.^[^ ^5^, ^6^ ^]^ Intrinsic to materials with first‐order transitions is a thermal hysteresis, which reduces the cyclic MCE. Nonetheless, it is suggested that this roadblock can be exploited as part of the multistimuli (uniaxial stress and field) cooling cycle.^[^ ^7^, ^8^, ^9^, ^10^ ^]^ Both approaches require a material with a row of tailored properties to achieve a large cyclic temperature change under reasonable field strengths. As such, an ideal material that combines all required properties has not yet been found. This motivates the search for a robust material design workflow, which can both enable the discovery of novel compounds and guide the improvement of already known materials.

High throughput (HTP) density functional theory (DFT) calculations have been successfully applied to screen for magnetic materials such as antiperovskites, transition metal permanent magnets, ⁠and magnetic topological materials.^[^ ^11^, ^12^, ^13^, ^14^ ^]^ In the context of magnetocaloric materials, Bocarsly et al. proposed a screening proxy for MCE materials based on the structural distortion between FM and nonmagnetic (NM) states.^[^ ^15^ ^]^ This approach seeks to identify materials with strong magnetovolume coupling in the vein of the Bean–Rodbell model and has been used to optimize the MCE of the MnCoGe MM'X (M/M’ = metal, X = main group element) system and of Mn‐Sb alloys.^[^ ^16^, ^17^, ^18^ ^]^ Such a proxy has been recently generalized to the MCE metric including both the magnetoelastic response and internal energy and is applied to screen for promising candidates.^[^ ^19^ ^]^ However, the proxy is unable to predict transition temperatures and it hardly captures the nature of relevant transitions in MCE materials. Furthermore, as noted by Guillou et al. there is no single driving mechanism for first‐order transitions in magnetocaloric systems, i.e., various mechanisms such as magnetostructural coupling, metamagnetism, and magnetovolume effects exist.^[^ ^20^ ^]^ Therefore, a natural starting point for a HTP screening of magnetocaloric materials should include both the description of the structural/volume changes and of the magnetic order. For example, structural transitions in Heusler alloys frequently occur between the tetragonal martensite and a metastable cubic austenite phase, described by the energy as a function of the c/a ratio—the Bain Path.^[^ ^21^ ^]^ Other compounds may have more subtle energy surfaces, for instance, in FeRh the exchange interactions and induced Rh moments are dependent on the spin excitations of the Fe sublattice, hence the energy difference of the antiferromagnetic (AFM) and FM states is dependent on temperature.^[^ ^22^ ^]^ Similarly, La‐Fe‐Si exhibits competing high and low magnetization states with different volumes, with both longitudinal and transversal excitations playing a role in the transition.^[^ ^23^ ^]^

The MM´X family of compounds crystallizes in three structural polymorphs, i.e., the orthorhombic *Pnma* (MgSrSi/TiNiSi‐type), the hexagonal nonpolar *P*6_3_/*mmc* (Ni_2_In‐type), and the hexagonal polar *P*6_3_ *mc* structures.^[^ ^24^ ^]^ The primary mechanism for large MCE in the MM´X family is a magnetostructural transition, which occurs between the *P*6_3_/*mmc* austenite and *Pnma* martensite phases, as demonstrated in the Mn_1−_ *_x_* Fe*_x_* NiGe_1−_ *_y_* Si*_y_* compound.^[^ ^25^ ^]^ Each of such polymorphic structures also exhibit a plethora of intriguing properties, such as superconductivity, nontrivial topological Dirac/Weyl/node‐line semimetals, thermoelectrics, ferroelectrics, transparent conductors, and permanent magnets.^[^ ^26^, ^27^, ^28^, ^29^, ^30^, ^31^, ^32^ ^]^ This wealth of technologically significant effects, combined with rich physics and versatile compositions indicates that the MM´X family is particularly suitable for HTP studies. Besides MCE, there are other applications driven by structural phase transitions such as antiferroelectrics, zero thermal expansion alloys, and shape memory effects.^[^ ^24^, ^33^, ^34^ ^]^

**Figure 1:**
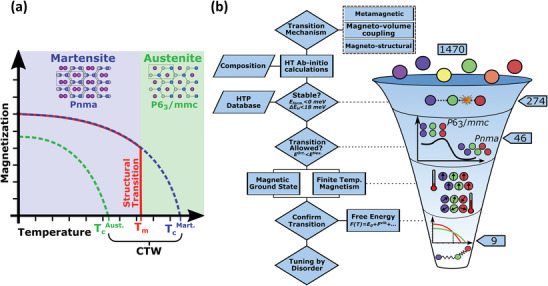
a) Schematic representation of a magnetostructural transition that occurs when the structural phase transition temperature *T*_m_ is within the Curie temperature window (CTW), taking the martensitic and austenitic phases of the MM´X compounds as an example. Both structures are showed overlayed with bonds connecting 2b and 2c positions, with the stuffing atom (purple) in‐between in the 2a position. b) HTP workflow to screen for novel magnetocaloric materials as applied to the MM´X compounds.

In particular, there are five ternary magnetic MM´X compounds, i.e., MnNiGe, MnNiSi, CoMnGe, CoMnSi, and FeMnSi, where a structural transition occurs between the low temperature *Pnma* and high temperature *P*6_3_/*mmc* structures.^[^ ^35^, ^36^, ^37^ ^]^ Unfortunately, for such stoichiometric MM´X compounds the transition occurs in the absence of magnetostructural coupling, thus without a sizeable MCE. In order to obtain magnetostructural coupling, it is essential that the structural transition temperature (*T*_m_) occurs within the Curie temperature window (CTW)—the temperature range that spans between the magnetic Curie temperature (T_c_) of the orthorhombic and hexagonal phases as depicted **Figure** **1**a. This scenario leads to a large magnetic entropy change, resulting in a large conventional MCE, since the structural transition occurs between states with different magnetization, e.g., low‐temperature FM to high‐temperature PM phases, as in the case of MnNiGe‐CoNiGe.^[^ ^38^ ^]^ Such a design concept can be extended to the inverse MCE, where the application of a magnetic field increases the entropy of the material. In this case, the structural transition would occur between a martensite phase without finite magnetization (such as AFM or PM) and a FM austenite, as is the case in the Ni‐Co‐Mn‐In Heusler system.^[^ ^39^ ^]^ Typical experimental efforts aim to both tune *T*_m_ to within the CTW and to control the magnetic ground state via isostructural doping.^[^ ^25^ ^]^ For instance, by alloying between MnNiSi (*T*_m_ = 1200 K) and FeNiGe, that is stable in the *P*6_3_/*mmc* (austenite) phase, the *T*_m_ is brought down to within the CTW, achieving a large MCE.^[^ ^40^ ^]^ Experimental examples of this approach include MnNiGe‐Al, Mn‐CrCoGe, MnNiGe‐Sn, and cosubstitution of Mn‐Fe and Si‐Ge in the MnNiGe system.^[^ ^25^, ^41^, ^42^, ^43^ ^]^ Recently, a computational approach to optimize the MCE by isostructural Al‐doping of Mn‐FeNiSi was also applied by Biswas et al.^[^ ^44^ ^]^

In this work, we report about the implementation of a computational workflow for identifying and optimizing potential magnetocaloric and magnetic shape‐memory alloys. We have applied this workflow to the screening of the MM´X family of ternary transition metal compounds. After validating the calculated stabilities and thermodynamic properties of experimental known cases, we elucidated how to quantify the underlying driving forces for the magnetostructural transition. Based on the concept of CTW, we identified nine potential candidates with suitable structural transitions, where tailoring of composition via isostructural doping suggests a practical route to engineer materials with large MCE. This paves the way for a more systematic design of magnetocaloric materials driven by first‐order phase transitions.

## 2 High‐Throughput Search

### 2.1 Search and Validation

In order to identity potential high‐performance magnetocaloric materials, the most essential descriptor is the existence of a magnetic first‐order transition. As shown in Figure 1a, for MM´X compounds, there is a martensitic transition between the hexagonal *P*6_3_/*mmc* and orthorhombic *Pnma* phases with a group‐subgroup relation and the corresponding transformation matrix (with an origin shift) reads^[^ ^45^ ^]^

(1) $$T_{\mathrm{Hex}.\to \mathrm{Ort}.}=\left(\begin{array}{cccc} 0 & -1 & 1 & 0\\ 0 & -1 & -1 & -0.5\\ 1 & 0 & 0 & 0\\ 0 & 0 & 0 & 1 \end{array}\right)$$

Correspondingly, the 2a, 2b, and 2c Wyckoff positions of the *P*6_3_/*mmc* are mapped to three distinct sites in the *Pnma* structure.^[^ ^46^ ^]^ From the geometrical point of view, the *P*6_3_/*mmc* lattice is composed of planes formed by the 2b and 2c positions, with the 2a sites in‐between the planes acting as stuffing atoms, as sketched in Figure 1a. Upon the transition to the *Pnma* phase, the planes buckle and the stuffing atoms are displaced, leading to a lower symmetry.^[^ ^24^ ^]^ A key question is the identification of the correct stuffing atom, i.e., the element in the 2a positions, since it can change during the DFT lattice relaxation procedure. In this work we identify the stuffing atoms based on which *Pnma* 4c positions best fit the corresponding *P*6_3_/*mmc* 2a positions, based on the group‐subgroup relation (see Table S1, Supporting Information). The variation of the element that is occupying the stuffing positions results in three distinct structures for each composition, with the occupation of the other sites being interchangeable. We systematically consider all three stuffing atom variations in our HTP search, with the ground state structure being selected in all follow‐up calculations.

After identifying the transition mechanism and associated crystal structures from the symmetry point of view, our resulting HTP workflow is depicted in Figure 1b. Starting from the three structural polymorphs, the thermodynamic stabilities for such variants are determined by evaluating the formation energies and the distances to the convex hull. Respectively, these criteria demonstrate stability in respect to decomposition toward elemental solids and into one or more competing structures, which along with the mechanical (via the elastic coefficients) and the dynamical (based on phonon spectra) stabilities are used to select thermodynamically (meta‐)stable compounds in HTP studies.^[^ ^11^ ^]^ Moreover, focusing on those compounds with possible martensitic transitions between the magnetic *P*6_3_/*mmc* and *Pnma* phases, the magnetic ground states and CTW are obtained via the calculation of Heisenberg exchange parameters from DFT, and follow‐up Monte Carlo (MC) simulations. Additionally, the structural transition temperature is estimated by evaluating the lattice contribution to the Gibbs free energy. To further optimize the compounds with most promising transitions, we also investigate possible substitutional paths for the novel and already known materials. Details of the computational details are reported in the Experimental Section. Our HTP calculations are carried out for 1470 compositions, where M is a 3d magnetic atom from (V, Cr, Ni, Mn, Fe, Co), M´ is chosen among (Li, Be, Sc, Y, Ti, Zr, Hf, V, Nb, Cr, Mo, W, Mn, Fe, Co, Ni, Cu, Zn), and X among (Bi, Sb, As, P, Pb, Sn, Ge, Si, In, Ga, Al, B, Zn, V, Ti, Mg). For each composition, DFT calculations are performed for the three polymorph structures discussed above, each one inclusive of three stuffing atom configurations. In total, 930 compounds are found to have negative formation energies, of which 274 have a distance to the convex hull below 18 meV atom^−1^. Among the stable compounds, we found that *Pnma* is the most frequently occurring ground state structure (250 compounds), followed by *P*6_3_/*mmc* (19) and then *P*6_3_ *mc* (6) (cf. Tables S2–S4, Supporting Information, respectively). Such results can be well validated by comparing with the known MM´X phases in the inorganic crystal structure database (ICSD) with 92 ternary *Pnma* (TiNiSi‐type) compounds in the chemical space considered in this work (see Table S5, Supporting Information).^[^ ^47^ ^]^ For such compounds, the average distance to the convex hull is 9 meV atom^−1^, while only seven compounds are above 18 meV atom^−1^, with the largest distance to the convex hull being MnCuAs at 189 meV atom^−1^. At the same time the average formation energy is −69 meV, with only MnCuAs having a positive value (10 meV), which we attribute to its AFM ordering.^[^ ^48^ ^]^ Regarding the hexagonal *P*6_3_/*mmc* (Ni_2_In‐type) structures present in the ICSD database, we find three ternary compounds reported to be synthesized at ambient pressure and without disorder: CoCrGe, CoNiSn, and CoFeGe. Our calculations attribute to all three a negative formation energy, but with a distance to the convex hull between 50 and 155 meV atom^−1^. Furthermore, to keep our HTP search computationally tractable we considered both the FM and NM states for each MM´X compound, while the screening of AFM ordering is performed later on for the compounds with possible magnetostructural coupling. Lastly, previous studies have noted that the cubic half‐Heusler (LiAlSi‐type) and *Pnma* TiNiSi‐type structures are two of the most common 1:1:1 compounds in the ABX, X = (Ni, Pd, Pt) composition.^[^ ^31^ ^]^ While we operate on a different chemical space, we nonetheless systematically include the cubic half‐Heusler structures in our determination of the distance to the convex hull, in particular considering all three possible variations of the half‐Heusler in the FM and NM states.

### 2.2 Stability Trends and Bonding

**Figure 2:**
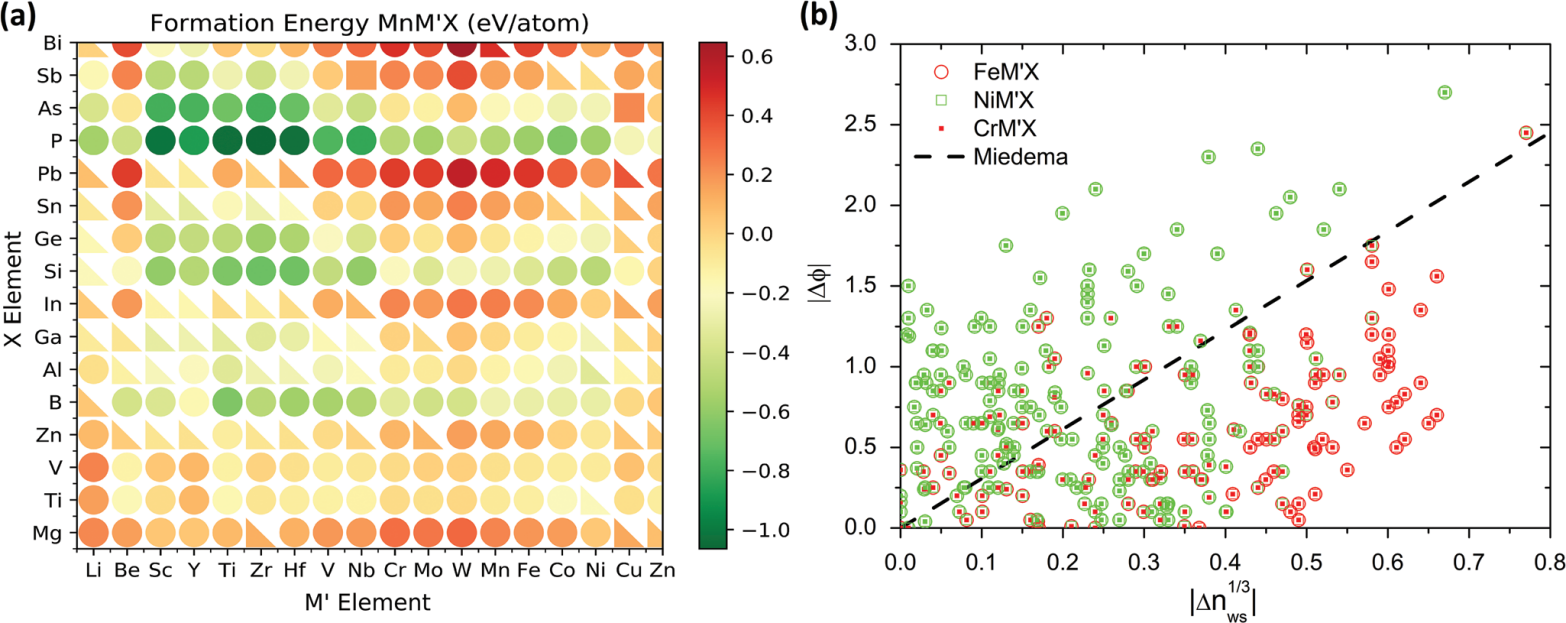
a) Heat map of the formation energy for the Mn‐M'X compounds, with each point is the most stable of the three structures. The circles are the *Pnma*, triangles are *P6_3_/mmc*, and squares are *P6_3_mc*. b) Plot of Miedema's theory stability criteria (below the dashed line the system is expected to be unstable) applied to the hypothetical M´X binary alloy. The circles represent Fe as the M atom and the square and square dot denote Ni and Cr, respectively, with negative formation energy in green and positive in red.

To shed light on the stability of the MM´X compounds, the formation energies for the Mn‐M´X systems are plotted in **Figure** **2**a. Obviously, the X elements that lead to the lowest formation energy are As, P, Ge, and Si, as expected from the experimentally known materials. This suggests that the X elements play an important role in the bonding and stability. In addition, the B‐ and Al‐based compounds also show low formation energies for several magnetic M elements. Regarding the trends of stability with respect to the M´ elements, it is apparent that compounds with early transition metal elements are the most stable. We note that a few stable MM´X are found with only d‐metal elements, namely when Ti and V act as the X element and late 3d metals act as the M´ (such as Co, Ni, and Cu), which is similar to the recently discovered all‐d‐metal Heusler Ni‐Co‐Mn‐Ti system.^[^ ^49^ ^]^ Such trends are similar for the MM´X compounds based on other 3d‐magnetic‐elements (cf. Figure S1, Supporting Information). Interestingly, the stability trends in MM´X systems can be well understood based on the bonding of the X and M´ elements in the orthorhombic MM´X systems. Landrum et al. explained the formation of the MM´Si phases using Miedema's semiempirical theory, pointing out that the stability of the ternary compounds depends on the hypothetical MM´ binary being stable.^[^ ^37^, ^50^ ^]^ Based on Miedema's theory, the formation energy of a binary compound is defined by two element specific parameters: the work function (*ϕ*) and electron density at the edge of the Wigner–Seitz cell (*n*_WS_). Specifically, a binary system is stable according to the condition

(2) $$\left|\Delta \phi \right|\geq \sqrt{9.4}\left|\Delta n_{\mathrm{ws}}^{1/3}\right|$$

The Δ*ϕ* parameter is related to the stability of bonds between atoms with different electronegativities and Δ*n*_WS_ accounts for the energy cost of bringing together elements with dissimilar electronic densities at the edges of their original Wigner–Seitz cell. Following Miedema's theory, it is observed that the stability of the MM´X compounds is mostly regulated by the bonding between the M´ (transition‐metals) atoms with the (mostly main group) X atoms to form M´X binaries for each magnetic M atom, as shown in Figure 2b (see also Figure S2, Supporting Information). For instance, all the X elements with more stable compounds from the set (P, As, Ge, Si, B, and Al) have large work functions. Conversely, it is the early transition metals (such as Sc, Y, Ti, Zr, Hf, and V) that lead to greater stability (through lower formation energies), due to their lower work functions that maximizes Δ*ϕ* and thus the electronegativity difference, which is desirable for covalent bonding. This also justifies the stability of hypothetical all‐d‐metal MM´X systems due to the lower work function of Ti and V compared to that of the late period elements for M´ such as Co, Ni, and Cu. Note that the magnetic M element is also expected to make strong bonds with X element; however, the application of Miedema's theory to the 3d magnetic M elements and X element pseudo‐binary was found to be less predictive, owing to the larger variety of M’ transition metal elements. To enable a better understanding of why the metal‐X bonding is responsible for stability, we perform crystal orbital Hamilton population (COHP) analysis for a few selected compounds shown in Table S6 in the Supporting Information.^[^ ^51^, ^52^, ^53^, ^54^ ^]^ COHP assigns a bonding and antibonding character to individual interactions from the electronic band‐structure, with integrated value at the Fermi energy providing the respective strength of the bond. It is observed that the bonds involving the X element and metals are the strongest, in particular the MX and M´X bonds, validating our analysis based on applying the Miedema's theory to the M´X binary. For instance, taking MnNiSi as an example, the Mn/Ni‐Si bonds are approximately three times stronger than the Mn‐Ni bonds. We also note that the respective hexagonal compounds show a slightly enhanced metal–metal bonding (cf. Table S6, Supporting Information).

## 3 Structural Transition

### 3.1 Candidate Selection

Turning now to the search for candidate materials with possible structural transitions, we follow the known cases (such as MnNiGe and MnNiSi) where the *Pnma* (*P*6_3_/*mmc*) phases are the low (high) temperature phases. Note that it is also possible that the *P*6_3_ *mc* structure acts as an austenite phase, as in the case of antiferroelectrics, which will be saved for future investigation⁠.^[^ ^20^ ^]^ Based on these considerations, the stable compounds are further filtered with the following sequence of criteria: i) *Pnma* is the stable ground state structure on or near the convex hull (<18 meV atom^−1^), leading to 240 ternary compounds, ii) the site occupation in the *P*6_3_/*mmc* austenite is defined by the *Pnma* structure to obey the Wyckoff splitting during the transitions (see Table S1, Supporting Information), iii) the *P*6_3_/*mmc* austenite phase obeys the formation energy criterion, reducing to 218 compositions, and iv) at least one phase has a magnetic moment of more than 0.25 µB per atom at *T* = 0. Applying such criteria results in 58 compounds. We point out that, crucially, all the compounds with known experimental transition, namely MnNiSi, MnNiGe, CoMnGe, CoMnSi, and FeNiSi, also satisfy the above conditions. The single exception is FeNiSi, which lies higher on the convex hull (48 meV atom^−1^) than our cutoff choice (see Table S8, Supporting Information), but it otherwise fulfils the selection conditions with possible phase transitions. Furthermore, compounds that fail the final criterion, i.e., where a transition is possible but are labeled nonmagnetic, can be potential candidates for nonmagnetic shape memory alloys and could likewise be tuned by isostructural substitution. As an example, NiVGe shows a small energy difference between austenite and martensite of only 28 meV atom^−1^. Upon comparing our calculations with experimental reports from ICSD, we find that some compounds such as CrNiP and FeTiP are concurrently indexed as both *Pnma* and Fe_2_P‐type, while the DFT total energy difference between such two structures amounts respectively to 8 and 19 meV atom^−1^, with the *Pnma* being more stable. Indeed, the Fe_2_P‐type is closely related to our three structural polymorphs and are present in the MM´Ge and MM´Si families.^[^ ^55^ ^]^ For this reason, we screen for the possibility of a hexagonal Fe_2_P‐type as the ground state and set aside those compounds where the *Pnma* structure is higher in energy than 18 meV atom^−1^ from the Fe_2_P‐type, as per our distance to convex hull cut‐off. Within the resulting set of materials that satisfy all these conditions, 22 compounds are present in the ICSD in the orthorhombic *Pnma* phase, while we find that only nine compounds are reported with another phase. This demonstrates the ability of our HTP search to reproduce experimental findings. With the exclusion of the latter mislabeled structures, our final dataset contains in total 46 compounds with a possible magnetostructural transition of interest (cf. Table S7, Supporting Information).

Beyond the thermodynamic stability, the mechanical and dynamical stabilities of the previously mentioned selected compounds are evaluated by calculating the elastic constants and phonon spectra.^[^ ^12^ ^]^ Hence, the elastic stability is established by applying a set of necessary and sufficient conditions to the elastic coefficients while dynamical stability is seen by the absence of imaginary modes (negative frequencies) in the phonon spectra.^[^ ^56^ ^]^ Such results are summarized in Table S7 in the Supporting Information.

We focus on the martensite since it is the *T* = 0 structure and should thus be stable from DFT, only two martensitic structures show dynamical instabilities (FeLiAs and FeHfAs) and two (FeLiGe and VNbGe) show mechanical instabilities, nonetheless we include them in the following analysis, noting that they are unlikely to be synthesized.

### 3.2 Finite Temperature Magnetism and Ground State

Moving on, we establish the finite temperature behavior of our novel MM´X alloys by calculating the CTW, the magnetic ground state ordering and *T*_m_. Our approach can be validated on the stoichiometric MM´X compounds with known structural transitions. In MnNiGe, the martensite ground state is spin‐spiral in the a‐b plane that changes into a spin spiral in the *a*‐axis at 185 K.^[^ ^57^ ^]^ Concurrently, there is a decrease of the spin moment of Mn from 2.75 to 2.2 µB, which complicates the description of the magnetic properties of MnNiGe. MnNiSi has a martensite *T*_c_ of 617 K and a local moment of 2.70 µB for Mn, for which we obtain 630 K and 2.76 µB in good agreement with experiments.^[^ ^58^ ^]^ Due to competing AFM and FM orderings, CoMnSi exhibits a metamagnetic first‐order transition (AFM to FM) and then a conventional magnetic transition (FM to PM) between 390 and 420 K, in good agreement with *T*_c_ of 460 K from our MC calculations, with the Mn magnetic moments being slightly overestimated by 0.34 µB (see Table S10, Supporting Information).^[^ ^59^, ^60^ ^]^ We also observe that when the unit cell is relaxed in an AFM ordering, it retains this behavior in our MC finite temperature calculations, demonstrating the ability of our calculations to capture the competition between AFM and FM states that originates the metamagnetic behavior. CoMnGe has a FM orthorhombic phase with an experimental *T*_c_ of 345 K, that is overestimated by 155 K from MC, despite a good agreement of local magnetic moments.^[^ ^60^, ^61^ ^]^ When using the disordered local moment (DLM) approach to mimic a PM state, the *T*_c_ becomes 330 K in agreement with experiment, that we attribute to the vanishing of Co moments when the Mn are in the DLM state.^[^ ^62^ ^]^ At the same time the hexagonal phase of the off‐stochiometric Co_0.92_Mn_1.07_Ge has a reported *T*_c_ of 245 K, while we obtain 125 K for stochiometric hexagonal CoMnGe.^[^ ^63^ ^]^ Following this satisfactory validation of our computational scheme, we apply it systematically to determine the Curie temperature for the selected candidate magnetostructural compounds of Table S9 in the Supporting Information.

As discussed above, some MM´X compounds adopt AFM magnetic ground states. This can have a significant influence on the MCE, but it is also an aspect of the material which can be tuned by isostructural substitution. For instance, in MnNiGe the martensite AFM ground state is undesirable because it leads to a phase transition to the PM austenite which cannot be easily manipulated by the external magnetic fields, as required in magnetocaloric solid‐state refrigeration applications. However, the substitution of Mn with Fe stabilizes the FM ordering and leads to a magnetostructural transition.^[^ ^25^ ^]^ On the other hand, the AFM‐FM transitions can still lead to a large inverse MCE.^[^ ^64^ ^]^ This motivates our further systematically screening of AFM states by adopting all possible symmetrically distinct collinear AFM configurations within selected supercell sizes (as detailed in the Experimental Section). In total, we find 20 orthorhombic and 24 hexagonal systems with an AFM ground states, as shown in Table S9 in the Supporting Information, along with the respective energy difference to the FM/NM configuration, with the lattice parameters and local moments shown in Tables S10 and S11 in the Supporting Information. The inclusion of AFM ordering in the total energy calculations also effects our outcome, lowering the energy of the phase an average of 30 meV atom^−1^, and thus affecting the martensitic transition temperature. Our Monte Carlo simulations provide an additional way to verify the theoretically predicted magnetic ordering by including finite temperature effects. We find an excellent agreement between the outcome of total energy DFT calculations and the configuration of the moments from the MC calculations (Table S9, Supporting Information). From the 46 compounds which have been examined in both phases, only eight cases showed disagreement among the two calculation approaches. We attribute the discrepancy to the small energy difference between the AFM and FM states in the specific compounds, which are mostly below 5 meV atom^−1^, and the fact that we limit ourselves to tractable supercell sizes. While we find that the DFT lattice parameters and local magnetic moments with the experimental values in literature (cf. Table S12, Supporting Information), the orthorhombic FeNiSi shows a more complex relation between structure, bonding, and magnetism. Neutron diffraction suggests the presence of only short‐range magnetic order with Fe moments of 0.96 µB.^[^ ^37^ ^]^ Other studies report no ferromagnetism down to room temperature.^[^ ^25^ ^]^ While DFT calculations in the FM setting point to a larger Fe moment of around 1.74 µB, together with relaxed lattice constants which deviate significantly from experimental reports (cf. Table S12, Supporting Information). However, if the system is instead assumed to be NM, the calculated lattice constants are in good agreement with experiments. It was noted by Landrum et al. that the experimental lattice constants of the MNiSi (with M as 3d element) series change sharply from Mn to Fe.^[^ ^37^ ^]^ This was attributed to a strong Fe‐Fe bonding which shortens the a‐axis lattice parameter. Our COHP analysis (see Table S13, Supporting Information) shows that in the FM DFT relaxed structure, the FeNiSi phase has weaker metallic Fe‐Fe (and Ni‐Ni) bonds, while displaying a stronger bonding between Fe‐Si and Ni‐Si than that obtained if we repeat the estimate using the experimental unit cell geometry, which is comparable with the lattice parameters within other MNiSi series. We interpret the outcome as indication of competition between the different types of bonds, which couples the lattice and magnetic degrees of freedom. The lack of strong FM ordering accounts for the different lattice constants of FeNiSi with respect to the rest of the MNiSi series.

### 3.3 Structural Transition Temperature Estimation

**Figure 3:**
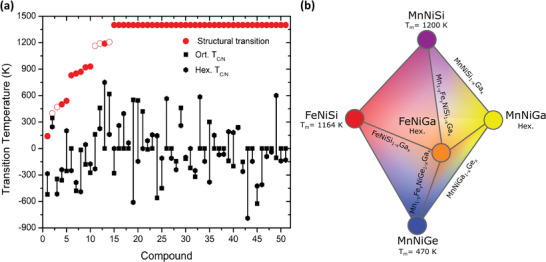
a) The left side plot shows the estimated *T*_m_ (red circles) from the QHA Debye model, with values for *T*_C/N_ of the hexagonal austenite (diamond marks) and orthorhombic martensite (squares). Negative values represent Néel temperature. Open symbols denote the five compounds known to have structural transitions, for which experimental data were used when available. Compounds that show no transition in the considered temperature range are labeled as 1400 K. b) The isostructural paths relating the MnNiGe, MnNiSi, and FeNiSi systems with the hexagonal Ga‐based compounds. The possible substitutions are indicated in text, with edges representing the replacement of one or more elements.

As discussed, the ultimate criterion to identify MM´X compounds for MCE applications is that *T*_m_ should fall inside of the CTW, thus being controllable by the applied magnetic fields. In principle, the martensitic transition temperature can be obtained by evaluating the free energy difference between the austenite and martensitic phases. We first consider the difference of total energies (Δ*E*_0_) between two phases as a descriptor to estimate the martensitic transition temperatures. This approximation proved successful in the case of Ni‐Mn‐X Heusler systems.^[^ ^65^ ^]^ However, the relation between the *E*_0_’s and the experimental *T*_m_ is less clear in the MM´X family, for instance, CoNiGe and CoMnSi have for instance similar Δ*E*_0_ but their *T*_m_ are 398 and 1190 K, respectively (Table S13, Supporting Information). In this regard, we found that the Quasi‐Harmonic Approximation (QHA) Debye model gives a more reasonable estimate, thanks to the accounting for vibrational contributions (cf. Figure S3, Supporting Information). We validate this approach using the transition temperature of the known ordered compounds (i.e., MnNiGe, MnNiSi, CoMnGe, CoMnSi, and FeMnSi) as reference. Taking MnNiSi as an example, the resulting free energies are shown in Figure S4 in the Supporting Information, where a phase transition temperature of 1200 K is expected from experiment and a value of 1100 K is obtained following the QHA Debye model. Therefore, we evaluate the *T*_m_ for all the 46 novel MM´X compounds using the QHA Debye model, along with the resulting CTW calculated from MC shown in **Figure** **3**a. The nine compounds listed in **Table** **1** exhibit a martensitic structural transition. The estimated transition temperature is above room temperature for almost all compounds (see Table 1). This is consistent with the range of structural transition temperatures for the known compounds, which lies between 398 and 1120 K (cf. Table S12, Supporting Information and Table 1). The largest difference in energy between phases of compounds with a predicted transition is 141 meV atom^−1^. However, there are also compounds with lower energy differences, which do not exhibit a transition (Table S14, Supporting Information). This indicates lack of a clear trend concerning what energy difference would guarantee a transition. While FeZrSb has a predicted *T*_m_ lower than room temperature (RT), due to the negligible energy difference of a few meV between the martensite and austenite it is hard to disambiguate the ground state structure. We highlight two other candidates with transitions MnTiGe and CrTiGe. The former has an AFM martensite and a FM austenite, while the latter has a NM martensite with a FM austenite with a high *T*_c_. If doping is used to optimize magnetostructural coupling, an inverse MCE would be expected. Interestingly, this is in contrast to known MM´X compounds where the martensite is FM and the PM/AFM austenite has no net magnetization. We note that there is no magnetostructural coupling in the known stoichiometric MM´X compounds with structural transitions, i.e., MnNiGe, CoMnGe, MnNiSi, CoMnSi, and FeNiSi, as observed in Figure 3a. This is due to the martensitic transitions occurring above the CTW and that the magnetic ground states involved can result in a CTW that cannot produce a net magnetization change, as attested by a negative CTW, e.g., AFM‐PM transition in MnNiGe. Thus, the stringent condition of the Tm falling inside the CTW implies the need for substitutional tuning that can control the structural transition temperature and maintain a wide CTW, as discussed below.

**Table 1:** DFT results for the novel compounds that are predicted to have a transition, showing the difference between the orthorhombic and hexagonal phases, along with their respective Curie and Néel (negative values) temperatures, magnetization difference between the phases (Δ*M^T^* ^=0^) at *T* = 0 and estimated *T*_m_. The experimental data for the compounds with known structural transitions is shown in parentheses

Phase	*E* ^ort.^ − *E* ^hex.^ [eV atom^−1^]	*T*_c/N_^Ort.^ [K]	*T*_c/N_^Hex.^ [K]	*T*_m_ [K]	*M* ^Ort.^[µB atom^−1^]	*M* ^Hex.^[µB atom^−1^]
FeZrSb	−0.002	−520	−285	140	Fe 2.1	Fe 1.9
CoMnGe	−0.042	500 (345^[^ ^66^ ^]^)	125 (245^[^ ^63^ ^]^)	311 (398–458^[^ ^35^ ^]^)	Mn 3.2 Co 0.7	Mn 2.7 Co 0.5
MnNiGe	−0.030	240 (−346^[^ ^57^ ^]^)	−515	455 (470–493^[^ ^35^, ^57^ ^]^)	Mn 3.1	Mn 3.0
FeLiGe	−0.031	−360	−240	500	Fe 2.0	Fe 2.1
MnTiGe	−0.094	−255	200	540	Mn 2.1	Mn 2.6
CrLiP	−0.141	0	−250	830	Cr 0.1	Cr 2.9
CrLiAs	−0.093	−480	−385	850	Cr 2.7	Cr 3.5
VLiSb	−0.109	−15	−490	870	V 1.1	V 2.6
FeNbGe	−0.065	−180	45	920	Fe 1.2	Fe 1.0
FeLiAs	−0.13	−275	−175	930	Fe 2.0	Fe 1.8/2.1
CrTiGe	−0.136	0	750	1190	Cr 0.0	Cr 2.3
FeNiSi	−0.038	160 (below RT^[^ ^37^ ^]^)	−230	405 (1164^[^ ^25^ ^]^)	Fe 1.7	Fe 1.9
CoMnSi	−0.037	460 (420^[^ ^59^ ^]^)	225	524 (1190^[^ ^67^ ^]^)	Mn 2.9 Co 0.7	Mn 2.5
MnNiSi	−0.060	630 (617^[^ ^58^ ^]^)	160	1147 (1206^[^ ^35^ ^]^)	Mn 2.8	Mn 2.5

### 3.4 Tuning by Disorder

In order to tailor the above candidates to achieve magnetostructural coupling and enhancing the MCE, we follow the overreaching design strategy of isostructural substitution that is used to establish a wide CTW and control the martensitic transition.^[^ ^25^ ^]^ We identify such isostructural substitution paths for both the newly predicted compounds (Table S15, Supporting Information) and the known cases (Table S16, Supporting Information), by determining related compositions where the hexagonal phase is preferred.

Starting with the five known compounds with structural transitions, this design paradigm can be validated by the experimentally realized cases, e.g., MnNiGe‐Al and Mn‐CrCoGe.^[^ ^41^, ^42^ ^]^ Another example is offered by Sn substitution as the X element, which has previously been applied in MnNiGe‐Sn.^[^ ^43^ ^]^ We further predict that Sn alloying would also be applicable in all the other known systems, given that both MnCoSn, MnNiSn and FeNiSn favor the hexagonal phase (cf. Table S16, Supporting Information). An examination of Table S16 in the Supporting Information shows that the hexagonal substitutional end point of the isostructural substitution (e.g., MnNiAl for MnNiGe‐Al and MnNiSn for MnNiGe‐Sn) does not have to be stable for the intermediate doped compounds to remain stable if they are close in composition to their stable parent phase.

We first highlight Ga‐based isostructural substitution in the known MnNiGe/MnNiSi and FeNiSi compounds, by tuning toward the hexagonal MnNiGa and FeNiGa phases, respectively, as sketched in Figure 3b. MnNiGa has been reported as synthesized in the hexagonal *P6_3_/mmc* structure, in line with the low distance to the convex hull from our HTP calculations.^[^ ^68^ ^]^ On the other hand, FeNiGa is likely unstable but nonetheless prefers the hexagonal phase. This allows control of the structural transition in FeNiSi*_x_* Ga_1−_ *_x_*_,_ provided that the Ga concentration is not high. Regarding magnetism, the martensite of FeNiSi is weakly FM, while MnNiSi is FM and MnNiGe is AFM, and we predict FeNiGa to be AFM with a Néel temperature of 450 K, while MnNiGa has a known *T*_c_ of 350 K. It has been noted that the magnetic behavior depends strongly on the Mn‐Mn distances that can be tuned by doping, allowing for the control of the CTW.^[^ ^25^ ^]^ Beyond this, there is the possibility for cosubstitution according to the formulations Fe*_y_* Mn_1−_ *_y_* NiSi*_x_* Ga_1−_ *_x_* and Fe*_y_* Mn_1−_ *_y_* NiGe*_x_* Ga_1−_ *_x_*, where the Fe‐Mn replacement can effectively control magnetism without detrimental effects to the optimized structural transition achieved by Ge/Si‐Ga doping. Along these lines an example of sizable MCE in Mn_0.4_Fe_0.6_NiSi_1−_ *_x_* Ga*_x_* has been reported by Chen et al. however MnNiGa‐Ge, MnNiSi‐Ga and FeNiSi‐Ga systems remain unexplored.^[^ ^69^ ^]^

As such, we confirm the effects of isostructural substitution in FeNiSi*_x_* Ga_1−_ *_x_*, MnNiSi*_x_* Ga_1−_ *_x_*, and MnNiGe*_x_* Ga_1−_ *_x_* by calculating the relative energies of the martensite and austenite as a function of composition using special quasi‐random structures (SQS) to treat disorder.^[^ ^70^ ^]^ This approach can be used to predict how the energy difference of the austenite and martensite changes with composition, thus serving to clarify how doping controls the *T*_m_. It also provides the range of stability of the martensite phase and thus the transition, as can be seen in Figure S5 in the Supporting Information for the case of Mn_1−_ *_x_* Fe*_x_* NiSi compared to experiment.

**Figure 4:**
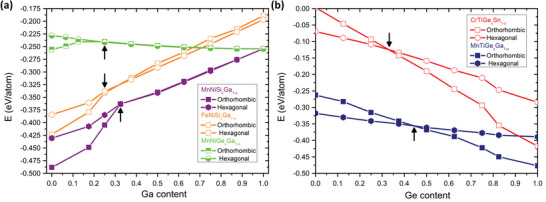
a) The formation energy for the orthorhombic (squares) and hexagonal structures (hexagons) as a function of composition, showing the effects of isostructural doping on the energy difference Ga‐Si substitution on the known FeNiSi (empty in orange) and MnNiSi (filled in purple) compounds and Ga‐Ge for MnNiGe (half‐filled in green). b): The results of the isostructural substitution for the MnTiGe*_x_* Ga_1−_ *_x_* (blue) and CrTiGe*_x_* Sn_1−_ *_x_* (red) novel compounds. The arrows indicate the crossover from the martensite to the austenite and the maximum threshold controlling the *T*_m_, while maintaining the transition.

In the FeNiSi_1−_ *_x_* Ga*_x_* and MnNiSi_1−_ *_x_* Ga*_x_* compounds, the austenite becomes the ground state for values of around *x* = 0.3 and *x* = 0.35, respectively, as shown in **Figure** **4**a. Thus, intermediate Ga content lowers the energy difference between austenite and martensite phases and enables the control of *T*_m_. At the same time, the formation energy of both compounds remains negative throughout the whole concentration range, which in principle allows for stable alloy formation. Likewise, inferring from the behaviour of MnNiGe*_x_* Ga_1−_ *_x_* in Figure 4a, the same Ga doping strategy is also viable in MnNiGe, while lowering the amount of expensive Ge. Notably, Fe‐Mn substitution in MnNiGe has been used to stabilize ferromagnetism in the AFM martensite of MnNiGe by doping toward hexagonal FeNiGe.^[^ ^25^ ^]^ As such, the Ge/Si‐Ga and Fe‐Mn substitutional paths in MnNiGe and MnNiSi, are validated by our SQS calculations and literature reports. Putting both paths together, as sketched in Figure 4b, the cosubstitution toward Fe*_y_* Mn_1−_ *_y_* NiSi*_x_* Ga_1−_ *_x_* and Fe*_y_* Mn_1−_ *_y_* NiGe*_x_* Ga_1−_ *_x_* can allow for the control of *T*_m_ while tuning the magnetic properties. Another possible pathway for MnNiSi and MnNiGe is to adopt Ti as the X element replacing Si/Ge, for instance, in the case of MnNiSi_1−_ *_x_* Ti*_x_*, *x* = 0.125 is sufficient to stabilize the hexagonal state while remaining stable (c.f., Figure S6, Supporting Information). Likewise, Cu substitution in MnNiGe and CoMnGe toward MnCuGe is also a promising approach. As shown in Figure S7 in the Supporting Information, only small amounts of Cu doping are needed to control *T*_m_. Since Ni and Co carry little magnetic moment compared to Mn, this alloying would not be detrimental to the magnetic properties, with the end point MnCuGe being magnetic. Regarding novel MM´X systems, we specifically discuss the isostructural substitution in two related compounds, MnTiGe and CrTiGe. Both systems are expected to exhibit an inverse MCE, if magnetostructural coupling is accomplished by lowering the *T*_m_. As previously discussed, in MnTiGe the austenite is the high magnetization phase due to the AFM ground state of the martensite. One can tune the energy difference between both phases through substitution toward hexagonal MnTiGa, as plotted in Figure 4b. This can proceed up to 45% of Ge, beyond which the austenite becomes the ground state. Crucially, orthorhombic MnTiGa is weakly magnetic, while the lower energy hexagonal ground state is magnetic with a *T*_c_ of 720 K from DFT. This preserves the CTW and thus the possibility of magnetostructural coupling and potentially enhancing the *T*_c_ of the austenite. In the case of CrTiGe, the substitutional end point compound, CrTiSn, shows similar magnetic properties with the martensite remaining NM and the austenite *T*_c_ being enhanced to around 900 K. We therefore expect that the wide CTW would be preserved over the substitutional range, up to alloying by around 33% Ge, where the martensite is no longer stable (cf. Figure 4b). This should allow for magnetostructural coupling with disorder.

## 4 Conclusion

In summary, we established a HTP workflow for computational design of novel MCE materials and applied it to the magnetostructural transition in the MM´X ternary compounds. From our initial screening we find 274 MM´X compounds that are thermodynamically stable in terms of formation energies and distances to the convex hull. Based on Miedema's theory, it is observed that the bonding between the M´‐X atoms is a key factor for stability, with X = (P, As, Ge, Si, B) elements being preferred due to their large values for the work function. Also considering the relative energies between the austenite and martensite phases, we identify 46 stable magnetic MM´X compounds with possible phase transitions. Further evaluation of the CTW and of the phase transition temperature based on the QHA Debye model allows to confirm that nine compounds exhibit a transition. Based on the concept of isostructural substitution, we identify doping strategies, such as alloying with Ga in the Mn‐FeNiSi and Mn‐FeNiGe systems, or with Ti and Cu doping in other compounds, which should realize magnetostructural coupling that is critical for magnetocaloric and magnetic shape‐memory applications.

## 5 Experimental Section

The stability screening was conducted using the Vienna ab initio simulation package (VASP) by means of an in‐house developed high‐throughput code.^[^ ^12^, ^71^, ^72^ ^]^ Trial structures were generated for all permutations of the Wyckoff positions for a given compound and then fully relaxed down to 10^−4^ eV as the structural convergence criteria, with the choice of the Perde‐Burke‐Ernzerhof (PBE) exchange‐correlation functional.^[^ ^73^ ^]^ We adopted VASP's automatic scheme that generates a Gamma‐centered Monkhorst–Pack grid, the *k*‐mesh density was set to 35, meaning the total number of *k*‐points in each direction is ≈35 multiplied by the reciprocal lattice vector and a cutoff energy of 550 eV for the plane waves basis expansion. To assess the stability, we considered the formation energy and distance to the convex hull, calculated from competing phases in our internal database. Furthermore, we consider as directly competing phases the half‐Heusler and LiGaGe‐type (*P*6_3_ *mc*) structures, with the latter being the polar equivalent to our *P*6_3_/*mmc* prototype. For each composition we also probed the three possible unique variations generated by switching the occupation of the stuffing atom site, and then selecting the lowest energy configuration.

The phonon calculations were carried out via VASP interfaced with the phonopy code.^[^ ^74^ ^]^ We used 2 × 2 × 2 and 2 × 2 × 1 supercells respectively for the *P*6_3_/*mmc* and *Pnma* phases. The *k*‐point mesh density was set to 55 in the VASP automatic mode and a cutoff 540 eV was employed. Elastic constants calculations were performed in the FM setting using the Elastic package to generate distorted structures and fit the respective stress values calculated by VASP.^[^ ^75^, ^76^ ^]^ We adopt a plane wave cut‐off of 700 eV, with a Gamma‐centered *k*‐mesh in the Monkhorst−Pack scheme with a *k*‐mesh density of 40. The Debye temperature is calculated from elastic constants by first estimating the speed of sound in the material under the averaging scheme of Anderson. The vibrational contribution to the free energy is obtained using the Debye model in the quasi‐harmonic approximation, i.e., at each volume point the vibrational lattice free energy is calculated for the given temperature and a Birch–Murnaghan fit is performed to find the minima of the free energy at each temperature.^[^ ^77^ ^]^

The estimation of the Curie temperature was performed using an in‐house MC Metropolis sampling code applied to the classical Heisenberg model, using exchange interactions obtained using DFT within a radius of four lattice parameters. The exchange interactions were calculated with the SPRKKR code by means of the magnetic force formula applied to the FM electronic structure reobtained using the PBE functional, an in‐house automation script was used to handle file generation.^[^ ^78^, ^79^, ^80^ ^]^ In particular, we used a spherical harmonics cut‐off of *l* = 3 and a *k*‐mesh density of about 2000 points per irreducible Brillouin zone wedge. For a few cases where electronic convergence was not achieved in SPRKKR, the JuKKR code was used at similar settings.^[^ ^81^ ^]^ The MC Metropolis sampling calculations were performed using a 10 × 10 × 10 supercell, with the Curie temperature being obtained by examining the Binder cumulant for FM systems and heat capacity peak for AFM cases. We cross‐validate the predicted ground state for selected compounds by comparing the energy of several colinear AFM configurations and the FM state. The AFM configurations were created by using the SUPERCELL code to automatically generate all possible symmetrically unique configurations with an equal number of antiparallel magnetic moments.^[^ ^82^ ^]^ For the hexagonal structures we consider 1 × 1 × 1, 1 × 1 × 2, 1 × 1 × 3, and 2 × 2 × 1 supercells, and in the *Pnma* structure 1 × 1 × 1, 2 × 1 × 1, 1 × 2 × 1, and 1 × 1 × 2. For disordered systems we employed SQS's constructed with the mcsqs code using 2 × 2 × 1, 2 × 1 × 2, and 1 × 2 × 2 structures for the orthorhombic structures and for the hexagonal systems we used 2 × 2 × 1, 2 × 1 × 2, 1 × 2 × 2, and 2 × 2 × 2 supercells.^[^ ^70^ ^]^

## Conflict of Interest

The authors declare no conflict of interest.

## Supporting information

Supporting Information

## Data Availability

The data that support the findings of this study are available from the corresponding author upon reasonable request.
