# Supplementary material for: High‐Throughput Design of Magnetocaloric Materials for Energy Applications: MM´X alloys
Source: Adv Sci (Weinh). 2023 Apr 20;10(17):2206772. doi: 10.1002/advs.202206772 (PMC10265063; doi:10.1002/advs.202206772)
Supplement: Supplementary file 1 — Supporting Information [file ADVS-10-2206772-s001.pdf]

ADVANCED SCIENCE
Open Access

Supporting Information

for *Adv. Sci.*, DOI 10.1002/advs.202206772

High-Throughput Design of Magnetocaloric Materials for Energy Applications: MM´X alloys

Nuno M. Fortunato, Andreas Taubel, Alberto Marmodoro, Lukas Pfeuffer, Ingo Ophale, Hebert Ebert, Oliver Gutfleisch and Hongbin Zhang**

Supplementary

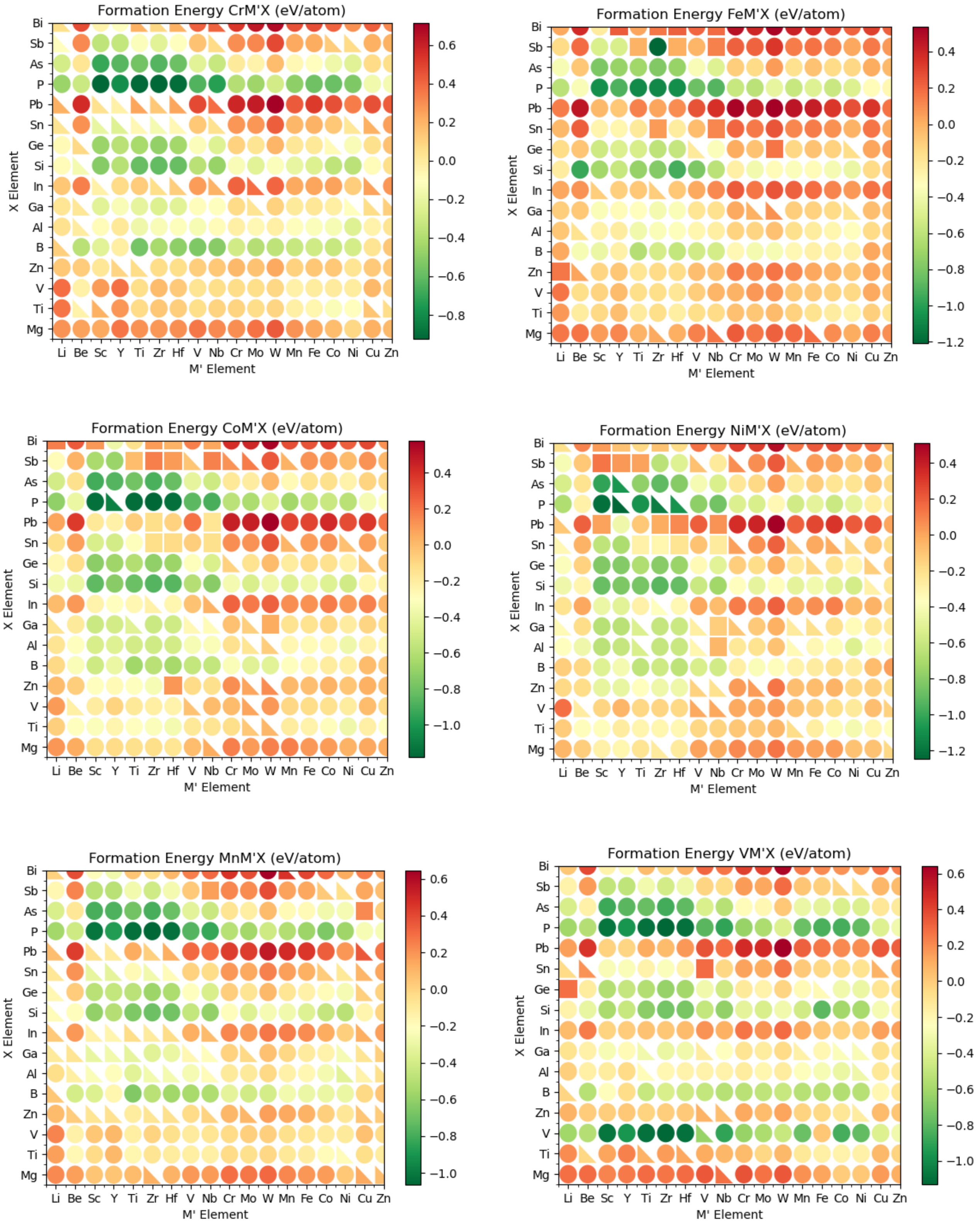

Figure S.1- Heat map of the formation energy for the pseudo-binary MM'X, for the lowest energy structure for the three polymorphs *Pnma (circle), P63/mmc (triangles) and P63mc(squares).*

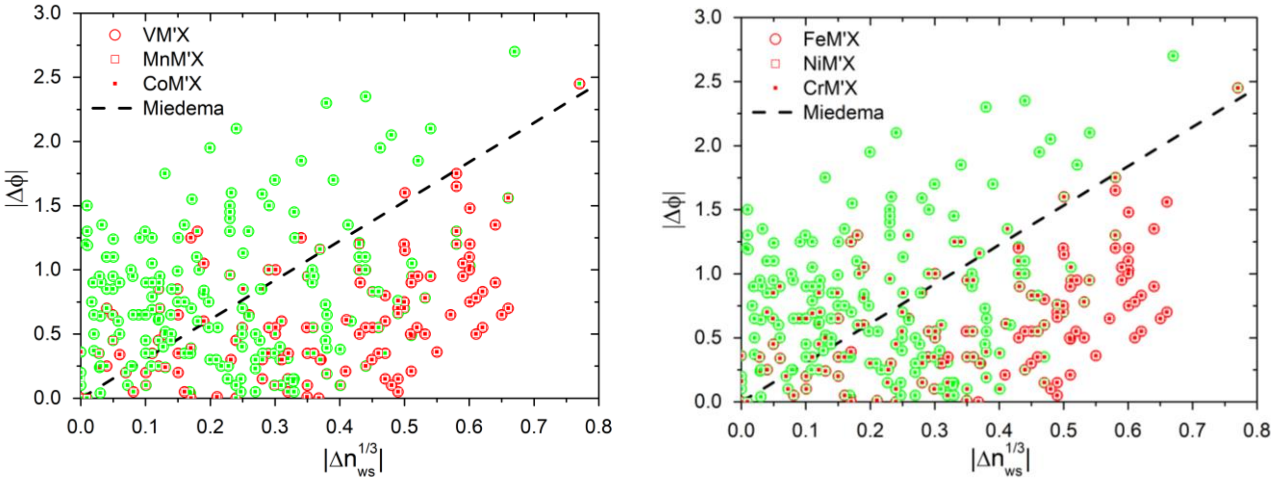

Figure S.2- Plots of Miedema theory stability criteria (dashed line) applied to the hypothetical M'X binary alloy alloy, red denotes positive formation energy and green negative (stable) formation energy.

Table S.1- Coordinates of the 2a Wyckoff positions (stuffing atom) in the sub-group basis, for both the original supergroup positions and the representative positions.

**2a Wyckoff of supergroup in subgroup basis**			**Representative positions in the subgroup for the respective 2a of the supergroup**		
0.000	0.750	0.750	-x	0.750	-z
0.500	0.250	0.250	0.5+x	0.250	0.5-z
0.000	0.250	0.250	x	0.250	z
0.500	0.750	0.750	0.5-x	0.750	0.5+z

Table S.2- Stable orthorhombic ternary compounds from HTP the search, that are not found in the ICSD.

**Form. Energy (eV/atom)**	**Dist. to convex hull (eV/atom)**	**Form. Energy (eV/atom)**	**Mag. Mom. (μB/atom)**
VBeAl	0.000	-0.157	0.000
CoScAl	0.000	-0.485	0.000
CoYAl	0.000	-0.446	0.001
CoZrAl	0.000	-0.512	0.003
CrVAl	0.013	-0.139	0.008
FeHfAl	0.000	-1.532	1.182
FeVAl	0.000	-1.485	0.974
NiHfAl	0.000	-0.599	0.000
NiScAl	0.000	-0.644	0.000
NiYAl	0.000	-0.611	0.000
VWAl	0.009	-0.135	0.002
CoLiAs	0.000	-0.495	0.003
CoMnAs	0.000	-0.271	1.028
CoNiAs	0.004	-0.228	0.001
CoScAs	0.003	-0.913	0.198
CoTiAs	0.000	-0.793	0.000
CoVAs	0.000	-0.412	0.000

CoZrAs	0.000	-0.888	0.000
CrLiAs	0.000	-0.283	0.058
CrNiAs	0.000	-0.234	0.999
FeHfAs	0.000	-0.718	0.090
FeLiAs	0.000	-0.369	0.475
FeTiAs	0.000	-0.692	0.073
FeVAs	0.000	-0.362	0.000
FeZrAs	0.000	-0.779	0.120
MnHfAs	0.000	-0.710	0.669
NiHfAs	0.000	-0.807	0.000
VHfAs	0.000	-0.787	0.000
MnLiAs	0.000	-0.382	0.853
NiLiAs	0.000	-0.547	0.000
VLiAs	0.000	-0.429	0.000
MnNiAs	0.000	-0.252	1.170
MnTiAs	0.000	-0.689	0.664
MnZrAs	0.000	-0.786	0.675
VNbAs	0.000	-0.538	0.000
NiScAs	0.000	-1.020	0.000
NiVAs	0.000	-0.411	0.001
NiZrAs	0.000	-0.906	0.001
VTiAs	0.000	-0.769	0.000
VZrAs	0.000	-0.859	0.000
CoBeB	0.000	-0.327	0.001
FeBeB	0.000	-0.372	0.157
MnBeB	0.000	-0.394	0.000
CoNbB	0.000	-0.552	0.004
CoYB	0.000	-0.395	0.002
FeMoB	0.000	-0.385	0.363
FeNbB	0.000	-0.522	0.580
MnMoB	0.000	-0.407	0.508
MnNbB	0.011	-0.513	0.368
MnWB	0.000	-0.391	0.516
NiNbB	0.000	-0.570	0.000
NiWB	0.000	-0.358	0.001
CoBeTi	0.000	-0.305	0.000
FeBeSi	0.000	-0.960	1.187
VBeGa	0.000	-0.165	0.000
MnBeP	0.003	-0.408	0.000
NiBeTi	0.000	-0.352	0.000
VBeP	0.014	-0.534	0.000
VBeSi	0.015	-0.347	0.000
CoLiBi	0.000	-0.020	0.014
CoCrGa	0.000	-1.897	1.023
CoHfGa	0.000	-0.509	0.001
CoScGa	0.000	-0.523	0.000
CoYGa	0.000	-0.496	0.003
CoZrGa	0.000	-0.528	0.000

CoLiGe	0.000	-0.260	0.001
CoNiGe	0.005	-0.219	0.004
CoTiGe	0.000	-0.607	0.004
CoHfTi	0.000	-0.352	0.000
CoLiP	0.000	-0.698	0.000
CoLiSb	0.000	-0.279	0.003
CoLiSn	0.000	-0.125	0.234
CoMoSi	0.000	-0.478	0.001
CoNiP	0.000	-0.578	0.001
CoNiSi	0.000	-0.502	0.007
CoScSb	0.000	-0.636	0.241
CoScTi	0.000	-0.318	0.002
CoYSi	0.000	-0.728	0.002
CoYTi	0.014	-0.244	0.001
CoTiZn	0.010	-0.300	0.005
CoZrTi	0.000	-0.315	0.000
CoYZn	0.010	-0.271	0.001
CoZrZn	0.000	-0.335	0.001
CrLiGe	0.000	-0.074	0.002
CrHfP	0.000	-0.890	0.353
CrLiP	0.000	-0.450	0.007
CrMnP	0.000	-0.481	0.599
CrVP	0.000	-0.628	0.000
CrTiSi	0.001	-0.589	0.000
NiCuTi	0.000	-0.311	0.000
FeHfGe	0.000	-0.579	0.001
FeLiGe	0.000	-0.156	0.607
FeMnGe	0.000	-0.131	1.653
FeNbGe	0.000	-0.351	0.118
FeScGe	0.005	-0.549	0.150
FeTiGe	0.010	-0.514	0.000
FeVHf	0.000	-0.205	0.000
FeVIn	0.000	-2.030	1.078
FeLiP	0.000	-0.604	0.000
FeLiSb	0.000	-0.149	0.764
FeLiSn	0.000	-0.021	0.766
FeMoSi	0.001	-0.394	0.001
FeNiP	0.016	-0.479	0.262
FeScP	0.000	-1.053	0.002
FeVP	0.000	-0.824	0.000
FeWP	0.000	-0.430	0.000
FeZrP	0.000	-1.080	0.000
FeZrSb	0.000	-1.208	0.464
FeVSc	0.015	-0.123	0.001
FeTiSi	0.009	-0.728	0.000
FeVSi	0.000	-0.798	0.515
FeVZr	0.000	-0.160	0.002
NiHfGa	0.000	-0.589	0.000

NiZrGa	0.000	-0.613	0.000
MnHfGe	0.000	-0.529	0.595
VHfGe	0.000	-0.605	0.000
NiLiGe	0.000	-0.383	0.000
MnNbGe	0.000	-0.379	0.679
MnTiGe	0.000	-0.477	0.529
VNbGe	0.000	-0.466	0.000
VTiGe	0.000	-0.551	0.001
VZrGe	0.000	-0.645	0.000
MnHfP	0.000	-1.019	0.652
NiHfSb	0.000	-0.512	0.000
NiHfZn	0.000	-0.446	0.000
VHfSi	0.000	-0.716	0.000
NiLiIn	0.000	-0.209	0.001
NiZnIn	0.014	-0.166	0.000
MnLiP	0.000	-0.543	0.572
MnLiSb	0.000	-0.164	1.089
NiLiP	0.000	-0.672	0.000
NiLiSb	0.000	-0.402	0.001
NiLiSi	0.000	-0.437	0.000
NiLiZn	0.001	-0.169	0.000
VLiP	0.000	-0.596	0.000
VLiSb	0.000	-0.177	0.000
NiYMg	0.006	-0.318	0.000
MnMoP	0.000	-0.544	0.000
MnNbSi	0.000	-0.600	0.654
MnScP	0.000	-1.005	0.533
MnTiP	0.000	-1.024	0.616
MnVP	0.000	-0.748	0.359
MnWP	0.003	-0.408	0.001
MnScSi	0.015	-0.602	0.630
MnTiSi	0.000	-0.651	0.469
NiMoSi	0.000	-0.483	0.000
VNbSi	0.000	-0.653	0.000
NiScTi	0.000	-0.358	0.000
NiScZn	0.000	-0.471	0.000
NiWSi	0.008	-0.355	0.000
NiZnSn	0.013	-0.188	0.000
NiTiZn	0.000	-0.365	0.000
NiZrTi	0.007	-0.312	0.004
NiYZn	0.000	-0.466	0.000
NiZrZn	0.000	-0.443	0.001
VScP	0.000	-1.072	0.000
VTiP	0.000	-1.083	0.000
VTiSi	0.000	-0.670	0.000

Table S.3- Stable $P6_3/mmc$ ternary compounds from our HTP search.

Form. Energy (eV/atom)	Dist. to convex hull (eV/atom)	Form. Energy (eV/atom)	Mag. Mom. (μB/atom)
NiTiAl	0.010	-0.541	0.000
NiYAs	0.000	-1.071	0.000
VBeTi	0.000	-0.037	0.000
NiLiBi	0.000	-0.197	0.001
CoYP	0.015	-1.082	0.045
CoVGa	0.007	-0.261	0.001
CrLiSb	0.000	-0.104	1.334
MnLiGa	0.000	-0.095	1.026
NiLiGa	0.000	-0.337	0.000
VTiGa	0.001	-0.309	0.000
MnNiGa	0.013	-0.254	1.088
MnLiGe	0.000	-0.166	1.126
MnLiSn	0.000	-0.080	1.135
NiLiPb	0.000	-0.112	0.001
NiLiSn	0.000	-0.329	0.000
VLiSn	0.000	-0.051	0.498
NiNbZn	0.008	-0.203	0.000
NiYP	0.000	-1.237	0.001
NiZrP	0.000	-1.158	0.000

Table S.4- Compounds from HTP that are stable in $P6_3mc$.

Form. Energy (eV/atom)	Dist. to convex hull (eV/atom)	Form. Energy (eV/atom)	Mag. Mom. (μB/atom)
CoHfSb	0.000	-0.543	0.001
CoNbSn	0.000	-0.170	0.001
CoZrSb	0.000	-0.625	0.000
CoHfZn	0.000	-0.345	0.002
FeLiZn	0.000	-1.997	0.002
NiNbGa	0.008	-0.354	0.000

Table S.5- Ternary compounds belonging to the TiNiSi-type present in the ICSD with the respective results from HTP search.

Form. Energy (eV/atom)	ICSD ID	Dist. to convex hull (eV/atom)	Form. Energy (eV/atom)	Mag. Mom. (μB/atom)
MnCuAs	72413	0.190	0.010	1.192
ScNiP	50990	0.000	-1.208	0.000
ZrCoP	49726	0.000	-1.172	0.001
TiCoP	624646	0.000	-1.149	0.000
ScCoP	624621	0.000	-1.143	0.007
HfCoP	623786	0.000	-1.140	0.000
ZrVP	39562	0.000	-1.122	0.001
HfVP	656389	0.000	-1.084	0.002
HfNiP	638712	0.005	-1.073	0.000
NiTiP	646165	0.000	-1.072	0.000
FeTiP	633111	0.000	-1.071	0.000
HfFeP	86280	0.000	-1.057	0.035
MnZrP	76095	0.000	-1.055	0.661
HfFeSi	632263	0.000	-0.931	0.004

NiZrSi	646693	0.000	-0.922	0.000
ZrCrP	626529	0.023	-0.915	0.185
HfNiSi	638723	0.000	-0.896	0.000
NbVP	645178	0.000	-0.890	0.000
ScNiSi	41800	0.000	-0.888	0.000
NbCoP	624292	0.000	-0.886	0.001
NbFeP	632794	0.000	-0.886	0.000
ZrCoSi	625144	0.000	-0.870	0.000
HfCoSi	623795	0.000	-0.859	0.001
NbNiP	645088	0.000	-0.846	0.000
TiNiSi	18188	0.000	-0.845	0.000
VCoP	624659	0.000	-0.840	0.046
ScCoSi	420415	0.000	-0.840	0.000
YNiSi	79598	0.000	-0.828	0.000
HfCoAs	406953	0.000	-0.819	0.001
ScNiGe	86365	0.000	-0.818	0.000
NbMnP	68280	0.000	-0.817	0.352
NiYGe	637440	0.000	-0.813	0.000
CoTiSi	625085	0.000	-0.811	0.003
NiZrGe	637451	0.000	-0.797	0.000
TiNiAs	611086	0.000	-0.776	0.000
FeZrSi	633674	0.000	-0.774	0.003
NiVP	646176	0.000	-0.770	0.000
HfNiGe	636577	0.000	-0.747	0.000
NbCoSi	624322	0.000	-0.731	0.001
NbNiSi	645107	0.000	-0.726	0.000
ScCoGe	600159	0.000	-0.723	0.000
CrNbP	53189	0.000	-0.720	0.483
ZrCoGe	623685	0.000	-0.712	0.004
MnZrSi	76236	0.000	-0.701	0.545
NiSnY	105379	0.000	-0.693	0.000
HfMnSi	638600	0.000	-0.688	0.506
ScFeSi	84203	0.000	-0.684	0.079
NiTiGe	53862	0.000	-0.676	0.000
HfCoGe	623439	0.000	-0.674	0.007
YCoGe	623669	0.000	-0.668	0.006
ScNiGa	8502	0.000	-0.661	0.000
NiYGa	634986	0.000	-0.653	0.000
ZrNiSb	408195	0.000	-0.647	0.000
CoMnP	41556	0.000	-0.642	0.994
NiScSn	105338	0.000	-0.629	0.000
CoMoP	2421	0.000	-0.620	0.000
NbFeSi	632827	0.000	-0.614	0.006
ZrFeGe	632166	0.000	-0.603	0.007
ZrCrSi	626850	0.053	-0.602	0.001
FeMoP	632646	0.000	-0.601	0.013
VCoSi	409847	0.000	-0.600	0.000
HfCrSi	626157	0.014	-0.591	0.001
NiVSi	646660	0.000	-0.589	0.005
MnZrGe	637130	0.000	-0.574	0.840
CoCrP	622489	0.000	-0.571	0.650
FeMnP	632538	0.000	-0.561	0.795
MnNiP	643093	0.000	-0.560	0.724
CrFeP	625922	0.000	-0.559	0.319

CoFeP	622955	0.000	-0.553	0.677
NbNiAs	610993	0.000	-0.537	0.000
CrNiP	626440	0.000	-0.521	0.882
NbCoAs	610089	0.000	-0.519	0.007
NbNiGe	255846	0.000	-0.502	0.000
YCoSn	601850	0.006	-0.498	0.086
MnNiSi	643132	0.000	-0.488	0.942
NbFeAs	610502	0.000	-0.479	0.001
ScCoSn	624977	0.000	-0.474	0.001
NbCoGe	623540	0.000	-0.467	0.002
YMnGe	97806	0.149	-0.453	0.617
MnCoSi	87314	0.001	-0.448	1.162
CoWP	624662	0.000	-0.443	0.000
YNiPb	427254	0.000	-0.440	0.000
MoCoB	42894	0.000	-0.440	0.001
CoWB	613390	0.000	-0.437	0.000
WFeB	614256	0.000	-0.372	0.347
CrNiSi	165255	0.091	-0.366	0.665
CoCrSi	622515	0.095	-0.350	0.333
VNiGe	637435	0.000	-0.337	0.014
VCoGe	623660	0.000	-0.310	0.002
GeMnNi	637013	0.000	-0.256	0.993
MnCuP	72411	0.174	-0.237	0.369
MnCoGe	623484	0.006	-0.193	1.245

Table S.6- Values of COHP for selected compounds, showing both the nearest-neighbour bonds and sum up to a cut-off of 4.5 Å for spin up and down channels. The more negative values imply greater stability.

	**Orthorhombic**					**Hexagonal**		
**Bond**	**-ICOHP 1**	**Dist. 1**	**-ICOHP 2**	**Dist. 2**	**Sum Up/Down**	**-ICOHP 1**	**Dist. NN**	**Sum Up/Down**
					**MnNiSi**			
Mn-Mn	0.07/0.15	3.00	0.05/0.11	3.11	0.26/0.58	0.24/0.48	2.56	0.54/0.98
Mn-Ni	0.12/0.20	2.72	0.11/0.20	2.74	0.81/1.35	0.17/0.26	2.62	1.06/1.63
Mn-Si	0.72/0.82	2.47	0.57/0.64	2.56	3.33/3.79	0.52/0.58	2.62	3.25/3.61
Ni-Si	0.83/0.79	2.28	0.76/0.73	2.31	3.38/3.25	0.83/0.80	2.28	3.32/3.12
Ni-Ni	0.14/0.15	2.58	0.02/0.02	3.56	0.46/0.53	0.00/0.00	3.43	0.12/0.13
Si-Si	0.19/0.14	3.37	0.12/0.09	3.56	1.24/0.97	0.17/0.15	3.43	1.44/1.27
					**MnNiGe**			
Mn-Mn	0.06/0.12	3.15	0.04/0.12	3.20	0.21/0.44	0.21/0.45	2.63	0.47/0.93
Mn-Ni	0.13/0.23	2.76	0.12/0.20	2.81	0.68/1.16	0.14/0.23	2.70	0.84/1.42
Mn-Ge	0.67/0.80	2.54	0.55/0.64	2.63	3.17/3.74	0.48/0.55	2.70	2.88/3.28
Ni-Ge	0.84/0.77	2.35	0.74/0.69	2.39	3.35/3.12	0.81/0.79	2.36	3.29/3.15
Ni-Ni	0.14/0.14	2.64	0.01/0.01	3.68	0.43/0.46	0.00/0.00	3.53	0.12/0.13
Ge-Ge	0.17/0.12	3.48	0.11/0.08	3.68	1.18/0.90	0.16/0.13	3.53	1.41/1.21
					**FeNiSi**			
Fe-Fe	0.22/0.42	2.55	0.02/0.03	3.75	0.51/0.93	0.21/0.49	2.47	0.49/1.05
Fe-Ni	0.14/0.22	2.59	0.13/0.20	2.70	0.84/1.29	0.15/0.23	2.59	0.95/1.47
Fe-Si	0.70/0.80	2.39	0.61/0.69	2.47	3.40/3.82	0.48/0.58	2.59	3.02/3.58

Ni-Si	0.80/0.77	2.31	0.69/0.68	2.36	3.39/3.23	0.83/0.79	2.28	3.53/3.29
Ni-Ni	0.1/0.1	2.71	0.01/0.01	3.87	0.36/0.38	0.01/0.00	3.36	0.14/0.12
Si-Si	0.26/0.23	3.20	0.26/0.23	3.25	1.57/1.38	0.19/0.16	3.36	1.54/1.34
**CoMnSi**								
Mn-Mn	0.17/0.27	2.54	0.02/0.02	3.69	0.49/0.27	0.00/0.04	3.52	0.27/0.07
Mn-Co	0.18/0.34	2.66	0.14/0.28	2.73	1.57/0.80	0.37/0.65	2.33	2.58/1.53
Mn-Si	0.82/0.90	2.37	0.70/0.78	2.45	3.66/3.31	0.46/0.51	2.68	2.387/2.52
Co-Si	0.88/0.87	2.30	0.77/0.78	2.35	3.47/3.45	0.40/0.40	2.68	2.36/2.38
Co-Co	0.17/0.27	2.54	0.02/0.02	3.69	0.80/0.53	0.00/0.01	3.52	0.03/0.06
Si-Si	0.17/0.14	3.43	0.10/0.07	3.69	0.94/1.18	1.17/1.14	2.65	2.39/2.52
**CoMnGe**								
Mn-Mn	0.05/0.09	3.11	0.04/0.08	3.24	0.36/0.19	0.20/0.42	2.64	0.87/0.45
Mn-Co	0.20/0.41	2.56	0.16/0.28	2.74	1.69/0.91	0.17/0.29	2.69	1.73/1.00
Mn-Ge	0.64/0.73	2.56	0.52/0.59	2.70	3.05/2.67	0.48/0.54	2.69	3.22/2.87
Co-Ge	1.32/1.31	2.13	0.88/0.86	2.26	3.68/3.70	0.88/0.86	2.35	3.54/3.57
Co-Co	0.03/0.03	3.28	0.01/0.03	3.45	0.29/0.21	0.01/0.01	3.54	0.19/0.15
Ge-Ge	0.22/0.19	3.39	0.14/0.11	3.73	1.09/1.29	0.16/0.14	3.54	1.20/1.35
**FeNiTi**								
Fe-Fe	0.25/0.43	2.52	0.02/0.02	3.93	0.57/0.94	---	---	---
Fe-Ni	0.20/0.28	2.50	0.13/0.18	2.73	0.79/1.09	---	---	---
Fe-Ti	0.46/0.50	2.66	0.44/0.48	2.69	2.91/3.19	---	---	---
Ni-Ti	0.55/0.52	2.49	0.44/0.43	2.59	2.63/2.57	---	---	---
Ni-Ni	0.13/0.13	2.71	0.01/0.01	3.82	0.36/0.36	---	---	---
Ti-Ti	0.3/0.33	3.10	0.07/0.07	3.93	1.29/1.36	---	---	---

Table S.7- Stability of phases of the dataset with possible magneto-structural coupling.

Phase	Orthorhombic				Hexagonal		
	Form. Energy (eV/atom)	Dist. to convex hull (eV/atom)	Dynamical	Mechanical	Form. Energy (eV/atom)	Dynamical	Mechanical
FeZrSb	-1.208	0.000	STABLE	STABLE	-0.019	UNSTB	UNSTB
FeLiGe	-0.156	0.000	STABLE	UNSTB	-0.12	UNSTB	STABLE
MnTiGe	-0.477	0.013	STABLE	STABLE	-0.389	UNSTB	STABLE
CrLiP	-0.450	0.000	STABLE	STABLE	-0.286	UNSTB	STABLE
CrLiAs	-0.283	0.000	STABLE	STABLE	-0.233	STABLE	STABLE
VLiSb	-0.177	0.000	STABLE	STABLE	-0.045	UNSTB	STABLE
FeNbGe	-0.351	0.000	STABLE	STABLE	-0.292	STABLE	STABLE
FeLiAs	-0.369	0.000	UNSTB	STABLE	-0.206	UNSTB	STABLE
CrTiGe	-0.418	0.017	STABLE	STABLE	-0.285	STABLE	STABLE
CrNbP	-0.720	0.000	STABLE	STABLE	-0.342	UNSTB	STABLE
VHfAs	-0.787	0.000	STABLE	STABLE	-0.322	UNSTB	STABLE
VZrAs	-0.859	0.000	STABLE	STABLE	-0.411	STABLE	STABLE
VTiP	-1.083	0.000	STABLE	STABLE	-0.591	STABLE	STABLE
MnHfP	-1.019	0.000	STABLE	STABLE	-0.656	UNSTB	STABLE
MnZrP	-1.055	0.000	STABLE	STABLE	-0.726	STABLE	STABLE
MnTiP	-1.024	0.000	STABLE	STABLE	-0.061	UNSTB	UNSTB
MnHfSi	-0.688	0.000	STABLE	STABLE	-0.462	UNSTB	STABLE
VNbGe	-0.466	0.000	STABLE	UNSTB	-0.233	UNSTB	STABLE
MnZrGe	-0.574	0.000	STABLE	STABLE	-0.419	UNSTB	STABLE
FeLiSb	-0.149	0.000	STABLE	STABLE	-0.055	UNSTB	STABLE
CrHfSi	-0.591	0.014	STABLE	STABLE	-0.336	UNSTB	STABLE
FeHfAs	-0.718	0.000	UNSTB	STABLE	-0.014	UNSTB	UNSTB

CoCrP	-0.571	0.000	STABLE	STABLE	-0.37	UNSTB	STABLE
CoFeP	-0.553	0.000	STABLE	STABLE	-0.353	UNSTB	STABLE
CrFeP	-0.559	0.000	STABLE	STABLE	-0.282	UNSTB	STABLE
CrMnP	-0.481	0.000	STABLE	STABLE	-0.25	UNSTB	STABLE
CrNiP	-0.521	0.000	STABLE	STABLE	-0.351	UNSTB	STABLE
CrTiSi	-0.589	0.001	STABLE	STABLE	-0.394	STABLE	STABLE
FeZrGe	-0.603	0.000	STABLE	STABLE	-0.482	STABLE	STABLE
FeMnP	-0.561	0.000	STABLE	STABLE	-0.359	UNSTB	STABLE
FeNbSi	-0.614	0.000	STABLE	STABLE	-0.469	UNSTB	STABLE
FeScP	-1.053	0.000	STABLE	STABLE	-0.814	STABLE	STABLE
FeZrSi	-0.774	0.000	STABLE	STABLE	-0.594	UNSTB	STABLE
MnNbP	-0.817	0.000	STABLE	STABLE	-0.512	UNSTB	STABLE
MnScP	-1.005	0.000	STABLE	STABLE	-0.703	UNSTB	STABLE
MnVP	-0.748	0.000	STABLE	STABLE	-0.523	UNSTB	STABLE
MnZrSi	-0.701	0.000	STABLE	STABLE	-0.492	UNSTB	STABLE
VScP	-1.072	0.000	STABLE	STABLE	-0.546	UNSTB	STABLE
VZrP	-1.122	0.000	STABLE	STABLE	-0.545	UNSTB	STABLE
MnNiP	-0.560	0.000	STABLE	STABLE	-0.364	UNSTB	STABLE
FeZrP	-1.080	0.000	STABLE	STABLE	-0.081	UNSTB	UNSTB
FeTiP	-1.071	0.000	STABLE	STABLE	-0.141	UNSTB	UNSTB
VLiAs	-0.429	0.000	STABLE	STABLE	-0.213	UNSTB	UNSTB
CrLiGe	-0.074	0.000	STABLE	STABLE	-0.069	STABLE	STABLE
FeBeSi	-0.960	0.000	STABLE	STABLE	-0.101	UNSTB	UNSTB
VTiGe	-0.551	0.000	STABLE	STABLE	-0.337	UNSTB	UNSTB

Table S.8- Stability and magnetic moments of the experimental MM'X phases with structural transition.

Phase	Orthorhombic			Hexagonal	
	Form. Energy (eV/atom)	Dist. to convex hull (eV/atom)	Mag. Mom. (μ_B/atom)	Form. Energy (eV/atom)	Mag. Mom. (μ_B/atom)
MnCoSi	-0.448	0.000	1.161	-0.413	0.890
MnCoGe	-0.193	0.006	1.245	-0.183	1.049
MnNiGe	-0.256	0.000	0.996	0.228	0.971
MnNiSi	-0.488	0.000	0.942	-0.430	0.824
FeNiSi	-0.423	0.048	0.576	-0.384	0.619

Table S.9- Predicted magnetic properties, ground state and critical temperatures of phases in the magneto-structural dataset

Phase	Orthorhombic					Hexagonal				
	Mag. State DFT	Mag. Mom. (μB/atom)	E^{AFM}-E^{FM} (eV/atom)	T_c (K)	State MC	Mag. State DFT	Mag. Mom. (μB/atom)	E^{AFM}-E^{FM} (eV/atom)	T_c (K)	State MC
FeZrSb	121	0.00	0.006	520	AFM	211	0.00	0.317	285	AFM
FeLiGe	FM	0.60	-0.015	360	AFM	221	0.00	0.007	240	AFM
MnTiGe	211	0.00	0.005	255	AFM	FM	0.71	-0.013	200	FM
CrLiP	NM	0.00	0.000	NM	NM	221	0.00	0.028	250	AFM
CrLiAs	112	0.00	0.046	480	AFM	113	0.00	0.006	385	AFM
VLiSb	111	0.00	0.005	15	AFM	FM	0.99	-0.005	490	AFM
FeNbGe	111	0.00	0.005	180	AFM	FM	0.00	-0.004	45	FM
FeLiAs	111	0.00	0.023	275	AFM	112	0.00	0.060	175	AFM
CrTiGe	NM	0.00	0.000	NM	NM	FM	0.67	-0.02	750	FM

CrNbP	211	0.00	0.000	280	AFM	NM	0.00	0.000	NM	NM
VHfAs	NM	0.00	0.000	NM	NM	FM	0.57	-0.006	260	FM
VZrAs	NM	0.00	0.000	NM	NM	FM	0.61	-0.005	395	FM
VTiP	NM	0.00	0.000	NM	NM	FM	0.35	-0.001	65	FM
MnHfP	FM	0.65	-0.006	550	FM	221	0.00	0.000	610	AFM
MnZrP	FM	0.66	-0.010	545	FM	112	0.00	0.010	145	AFM
MnTiP	FM	0.61	-0.005	420	FM	NM	0.00	0.000	NM	NM
MnHfSi	211	0.00	0.006	10	AFM	FM	0.76	-0.004	90	FM
VNbGe	NM	0.00	0.000	NM	NM	FM	0.30	-0.002	155	FM
MnZrGe	112	0.00	0.016	560	AFM	FM	0.95	-0.003	145	FM
FeLiSb	121	0.00	0.027	450	AFM	112	0.00	0.018	110	AFM
CrHfSi	NM	0.00	0.000	NM	NM	FM	0.69	-0.027	565	FM
FeHfAs	NM	0.07	0.000	NM	NM	112	0.00	0.003	110	AFM
CoCrP	111	0.00	0.004	145	AFM	112	0.00	0.029	240	AFM
CoFeP	FM	0.68	-0.028	460	FM	FM	0.54	-0.007	260	FM
CrFeP	111	0.00	0.032	115	AFM	111	0.00	0.032	100	AFM
CrMnP	111	0.00	0.032	220	AFM	NM	0.00	0.000	NM	NM
CrNiP	111	0.00	0.002	320	AFM	FM	1.00	-0.007	250	AFM
CrTiSi	NM	0.00	0.000	NM	NM	FM	0.64	-0.030	585	FM
FeZrGe	NM	0.00	0.000	NM	NM	221	0.00	0.011	145	AFM
FeMnP	112	0.00	0.017	300	FM	112	0.00	0.036	380	AFM
FeNbSi	NM	0.00	0.000	NM	NM	FM	0.30	-0.007	150	FM
FeScP	NM	0.00	0.000	NM	NM	221	0.00	0.003	70	AFM
FeZrSi	NM	0.00	0.000	NM	NM	221	0.00	0.003	65	AFM
MnNbP	211	0.00	0.006	140	AFM	FM	0.63	-0.016	190	FM
MnScP	211	0.00	0.007	200	AFM	112	0.00	0.001	180	FM
MnVP	211	0.00	0.004	240	FM	FM	0.92	-0.010	235	FM
MnZrSi	211	0.00	0.010	150	AFM	112	0.00	0.091	260	AFM
VScP	NM	0.00	0.000	NM	NM	113	0.00	0.032	790	AFM
VZrP	NM	0.00	0.000	NM	NM	FM	0.43	-0.002	145	AFM
MnNiP	FM	0.73	-0.003	625	FM	221	0.00	0.022	425	AFM
FeZrP	NM	0.00	0.000	NM	NM	112	0.00	0.004	410	AFM
FeTiP	NM	0.00	0.000	NM	NM	112	0.00	0.007	90	AFM
VLiAs	NM	0.00	0.000	NM	NM	112	0.00	0.014	40	AFM
CrLiGe	211	0.00	0.010	105	AFM	FM	1.01	-0.008	600	FM
FeBeSi	NM	0.00	0.000	NM	NM	221	0.00	0.162	140	AFM
VTiGe	NM	0.00	0.000	NM	NM	221	0.30	0.007	130	AFM

* All AFM states converged to FM.

Table S.10- Predicted magnetic properties, ground state and critical temperatures of phases in the magneto-structural dataset

Phase	Stuffing atom	Lattice Param. (Å)	Mag. Mom. (μB/atom)	Crystallographic Coordinates (arb.)
FeZrSb	Fe	7.693<br>5.670	0 0 0 0 0 0 0 0 -1.9 -1.9 -1.9 -1.9 1.9 1.9 1.9 1.9 0 0 0 0 0 0 0 0.0	0.198,0.397,0.25 0.302,0.103,0.75 0.198,0.897,0.25 0.302,0.603,0.75 0.698,0.397,0.25 0.802,0.103,0.75 0.698,0.897,0.25 0.802,0.603,0.75 0,0,0.5 0,0.5,0.5 0.5,0,0 0.5,0.5,0 0,0,0 0,0.5,0 0.5,0,0.5 0.5,0.5,0.5 0.157,0.315,0.75 0.343,0.185,0.25 0.157,0.815,0.75 0.343,0.685,0.25 0.657,0.315,0.75 0.843,0.185,0.25 0.657,0.815,0.75 0.843,0.685,0.25
FeLiGe	Li	8.488<br>5.062	0 0 0 0 0 0 0 0 -2.1 -2.1 -2.1 -2.1 2.1 2.1 2.1 2.1 0 0 0 0 0 0 0 0	0.163,0.338,0.25 0.337,0.162,0.75 0.163,0.838,0.25 0.337,0.662,0.75 0.663,0.338,0.25 0.837,0.162,0.75 0.663,0.838,0.25 0.837,0.662,0.75 0.172,0.828,0.75 0.328,0.672,0.25 0.672,0.328,0.75 0.828,0.172,0.25 0.172,0.328,0.75 0.328,0.172,0.25 0.672,0.828,0.75 0.828,0.672,0.25

				0,0,0 0,0,0.5 0,0.5,0 0,0.5,0.5 0.5,0,0 0.5,0,0.5 0.5,0.5,0 0.5,0.5,0.5
MnTiGe	Ti	4.302 5.390	-0.4 -0.4 2.6 2.6 0 0	0,0,0.25 0,0,0.75 0.333,0.667,0 0.667,0.333,0.5 0.333,0.667,0.5 0.667,0.333,0
CrLiP	Li	8.403 5.138	2.9 2.9 2.9 2.9 -2.9 -2.9 -2.9 -2.9 0 0 0 0 0 0 0 0 0 0 0 0 0 0 0 0	0.167,0.333,0.25 0.333,0.167,0.75 0.667,0.833,0.25 0.833,0.667,0.75 0.167,0.833,0.25 0.333,0.667,0.75 0.667,0.333,0.25 0.833,0.167,0.75 0,0,0 0,0,0.5 0,0.5,0 0,0.5,0.5 0.5,0,0 0.5,0,0.5 0.5,0.5,0 0.5,0.5,0.5 0.167,0.333,0.75 0.333,0.167,0.25 0.167,0.833,0.75 0.333,0.667,0.25 0.667,0.333,0.75 0.833,0.167,0.25 0.667,0.833,0.75 0.833,0.667,0.25
CrLiAs	Li	4.395 16.708	3.4 3.5 3.5 -0.1 -0.1 -0.1 0.1 0.1 0.1 -3.5 -3.5 -3.4 0 0 0 0 0 0	0.333,0.667,0.25 0.667,0.333,0.064 0.667,0.333,0.436 0.333,0.667,0.093 0.667,0.333,0.25 0.333,0.667,0.407 0.667,0.333,0.593 0.333,0.667,0.75 0.667,0.333,0.907 0.333,0.667,0.564 0.333,0.667,0.936 0.667,0.333,0.75 0,0,0 0,0,0.165 0,0,0.335 0,0,0.5 0,0,0.665 0,0,0.835
VLiSb	Li	4.762 5.806	0 0 2.6 2.6 -0.1 -0.1	0,0,0.25 0,0,0.75 0.333,0.667,0 0.667,0.333,0.5 0.333,0.667,0.5 0.667,0.333,0
FeNbGe	Nb	4.103 6.107	-0.1 -0.1 1.0 1.0 0 0	0,0,0.219 0,0,0.719 0.333,0.667,0.063 0.667,0.333,0.563 0.333,0.667,0.468 0.667,0.333,0.968
FeLiAs	Li	3.930 12.760	0 0 0 0 0 0 -1.8 -2.1 -2.0 2.0 2.1 1.8 0 0 0 0 0 0	0,0,0 0.333,0.667,0.090 0.667,0.333,0.265 0.333,0.667,0.426 0.667,0.333,0.574 0.333,0.667,0.735 0.667,0.333,0.910 0.667,0.333,0.419 0.333,0.667,0.880 0.667,0.333,0.764 0.333,0.667,0.235 0.667,0.333,0.120 0.333,0.667,0.581
CrTiGe	Ti	4.348 5.348	-0.3 -0.3 2.3 2.3 0 0	0,0,0 0,0,0.5 0.333,0.667,0.75 0.667,0.333,0.25 0.333,0.667,0.25 0.667,0.333,0.75
CrNbP	Nb	8.194 5.747	0 0 0 0 0 0	0,0,0.222 0,0,0.722 0.333,0.666,0.057 0.333,0.666,0.472 0.666,0.333,0.972
VHfAs	Hf	4.511 5.646	0 0 1.5 1.5 0 0	0,0,0 0,0,0.5 0.333,0.667,0.75 0.667,0.333,0.25 0.333,0.667,0.25 0.667,0.333,0.75
VZrAs	Zr	4.529 5.696	0 0 1.6 1.6 0 0	0,0,0 0,0,0.5 0.333,0.667,0.75 0.667,0.333,0.25 0.333,0.667,0.25 0.667,0.333,0.75
VTiP	Ti	4.263 5.144	0.1 0.1 0.8 0.8 0 0	0,0,0 0,0,0.5 0.333,0.667,0.75 0.667,0.333,0.25 0.333,0.667,0.25 0.667,0.333,0.75
MnHfP	Hf	4.005 6.278	-0.1 -0.1 1.3 1.3 0 0	0,0,0.242 0,0,0.742 0.333,0.667,0.045 0.667,0.333,0.545 0.333,0.667,0.464 0.667,0.333,0.964
MnZrP	Zr	7.722 7.170	1.8 1.8 1.8 1.8 -1.8 -1.8 -1.8 -1.8 -0.1 -0.1 0.1 0.1 0.1 0.1 -0.1 -0.1 0 0 0 0 0 0 0 0.0	0.167,0.333,0.75 0.333,0.167,0.25 0.667,0.833,0.75 0.833,0.667,0.25 0.167,0.833,0.75 0.333,0.667,0.25 0.667,0.333,0.75 0.833,0.167,0.25 0,0,0 0,0,0.5 0,0.5,0 0,0.5,0.5 0.5,0,0 0.5,0,0.5 0.5,0.5,0 0.5,0.5,0.5 0.167,0.333,0.25 0.333,0.167,0.75 0.167,0.833,0.25 0.333,0.667,0.75 0.667,0.333,0.25 0.833,0.167,0.75 0.667,0.833,0.25 0.833,0.667,0.75
MnTiP	Ti	4.00 5.432	0 0 0 0 0 0	0,0,0 0,0,0.5 0.333,0.666,0.649 0.666,0.333,0.351 0.333,0.666,0.177 0.666,0.333,0.823
MnHfSi	Hf	4.217 6.003	-0.1 -0.1 2.4 2.4 0 0	0,0,0 0,0,0.5 0.333,0.667,0.75 0.667,0.333,0.25 0.333,0.667,0.25 0.667,0.333,0.75
VNbGe	Nb	4.456 5.396	-0.1 -0.1 0.9 0.9 0 0	0,0,0 0,0,0.5 0.333,0.667,0.75 0.667,0.333,0.25 0.333,0.667,0.25 0.667,0.333,0.75
MnZrGe	Zr	4.386 6.003	-0.1 -0.2 2.9 2.9 0 0	0,0,0 0,0,0.5 0.333,0.667,0.75 0.667,0.333,0.25 0.333,0.667,0.25 0.667,0.333,0.75
FeLiSb	Li	4.465 11.535	0 0 0 0 -2.3 -2.3 2.3 2.3 0 0 0 0	0.333,0.667,0.116 0.667,0.333,0.384 0.333,0.667,0.616 0.667,0.333,0.884 0.333,0.667,0.856 0.667,0.333,0.644 0.333,0.667,0.356 0.667,0.333,0.144 0,0,1 0,0,0.25 0,0,0.5 0,0,0.75
CrHfSi	Hf	4.308 5.839	-0.1 -0.2 2.2 2.2 0 0	0,0,0 0,0,0.5 0.333,0.667,0.75 0.667,0.333,0.25 0.333,0.667,0.25 0.667,0.333,0.75
FeHfAs	Hf	4.444 11.442	0 0 0 0 1.6 1.6 0.2 0.2 -0.2 -0.2 -1.6 -1.6	0,0,1 0,0,0.25 0,0,0.5 0,0,0.75 0.333,0.667,0.877 0.667,0.333,0.623 0.333,0.667,0.129 0.667,0.333,0.371 0.333,0.667,0.629 0.667,0.333,0.871 0.333,0.667,0.377 0.667,0.333,0.123
CoCrP	Cr	3.779 11.427	2.4 2.4 -2.4 -2.4 0.1 -0.1 0.1 -0.1 0 0 0 0	0,0,0.132 0,0,0.632 0,0,0.382 0,0,0.882 0.333,0.667,0.021 0.667,0.333,0.271 0.333,0.667,0.521 0.667,0.333,0.771

				0.333,0.667,0.222 0.667,0.333,0.472 0.333,0.667,0.722 0.667,0.333,0.972
CoFeP	Fe	3.688 5.773	1.6 1.7 0 0 0 0	0,0,0.284 0,0,0.784 0.333,0.667,0.039 0.667,0.333,0.539 0.333,0.667,0.426 0.667,0.333,0.926
CrFeP	Cr	3.793 16.646	1.8 1.8 1.8 -1.8 -1.8 -1.8 0.2 -0.2 0.2 -0.2 0.2 -0.2 0 0 0 0 0 0	0,0,0.079 0,0,0.413 0,0,0.746 0,0,0.246 0,0,0.579 0,0,0.913 0.333,0.667,0.018 0.667,0.333,0.184 0.333,0.667,0.351 0.667,0.333,0.518 0.333,0.667,0.684 0.667,0.333,0.851 0.333,0.667,0.153 0.667,0.333,0.320 0.333,0.667,0.486 0.667,0.333,0.653 0.333,0.667,0.820 0.667,0.333,0.986
CrMnP	Cr	3.867 5.381	0 0 0 0 0 0	0.000,0.000,0.205 0.000,0.000,0.705 0.333,0.667,0.068 0.667,0.333,0.568 0.333,0.667,0.477 0.667,0.333,0.977
CrNiP	Cr	3.763 6.111	2.9 2.9 0.1 0 -0.1 -0.1	0,0,0.268 0,0,0.768 0.333,0.667,0.042 0.667,0.333,0.542 0.333,0.667,0.440 0.667,0.333,0.940
CrTiSi	Ti	4.258 5.282	-0.2 -0.2 2.1 2.1 0 0	0,0,0.25 0,0,0.75 0.333,0.667,0 0.667,0.333,0.5 0.333,0.667,0.5 0.667,0.333,0
FeZrGe	Zr	8.367 6.439	0 0 0 0 0 0 0 0 -1.8 -1.8 -1.8 -1.8 1.8 1.8 1.8 1.8 -0.1 0 0.1 0 0.1 0 - 0.1 0	0.167,0.333,0.466 0.333,0.167,0.966 0.167,0.833,0.466 0.333,0.667,0.966 0.667,0.333,0.466 0.833,0.167,0.966 0.667,0.833,0.466 0.833,0.667,0.966 0.167,0.833,0.048 0.333,0.667,0.548 0.667,0.333,0.048 0.833,0.167,0.548 0.167,0.333,0.048 0.333,0.167,0.548 0.667,0.833,0.048 0.833,0.667,0.548 0,0,0.236 0,0,0.736 0,0.5,0.236 0,0.5,0.736 0.5,0,0.236 0.5,0,0.736 0.5,0.5,0.236 0.5,0.5,0.736
FeMnP	Mn	3.782 11.295	0 0 0 0 -2.8 -2.8 0.3 -0.3 0.3 -0.3 2.8 2.8	0.333,0.667,0.223 0.667,0.333,0.473 0.333,0.667,0.723 0.667,0.333,0.973 0,0,0.381 0,0,0.881 0.333,0.667,0.021 0.667,0.333,0.271 0.333,0.667,0.521 0.667,0.333,0.771 0,0,0.131 0,0,0.631
FeNbSi	Nb	4.028 6.010	0 0 0.8 0.9 0 0.0	0,0,0.222 0,0,0.722 0.333,0.667,0.061 0.667,0.333,0.561 0.333,0.667,0.468 0.667,0.333,0.968
FeScP	Sc	7.930 6.321	0 0 0 0 0 0 0 0 0 0 0 0 0 0 0 0 -1.4 -1.4 -1.4 -1.4 -1.4 1.4 1.4 1.4 1.4	0,0,0.246 0,0,0.746 0,0.5,0.246 0,0.5,0.746 0.5,0,0.246 0.5,0,0.746 0.5,0.5,0.246 0.5,0.5,0.746 0.167,0.333,0.462 0.333,0.167,0.962 0.167,0.833,0.462 0.333,0.667,0.962 0.667,0.333,0.462 0.833,0.167,0.962 0.667,0.833,0.462 0.833,0.667,0.962 0.333,0.667,0.542 0.667,0.333,0.042 0.667,0.833,0.042 0.833,0.667,0.542 0.167,0.333,0.042 0.333,0.167,0.542 0.167,0.833,0.042 0.833,0.167,0.542
FeZrSi	Zr	8.190 6.382	0 0 0 0 0 0 0 0 -1.4 -1.4 -1.4 -1.4 1.4 1.4 1.4 1.4 -0.1 -0.1 0 0 0 0 0.1 0.1	0.167,0.333,0.470 0.333,0.167,0.970 0.167,0.833,0.470 0.333,0.667,0.970 0.667,0.333,0.470 0.833,0.167,0.970 0.667,0.833,0.470 0.833,0.667,0.970 0.333,0.667,0.541 0.667,0.333,0.041 0.667,0.833,0.041 0.833,0.667,0.541 0.167,0.333,0.041 0.333,0.167,0.541 0.167,0.833,0.041 0.833,0.167,0.541 0,0,0.238 0,0,0.738 0,0.5,0.238 0,0.5,0.738 0.5,0,0.238 0.5,0,0.738 0.5,0.5,0.238 0.5,0.5,0.738
MnNbP	Nb	4.005 5.920	-0.1 -0.1 1.0 1.0 0 0	0,0,0.220 0,0,0.720 0.333,0.667,0.063 0.667,0.333,0.563 0.333,0.667,0.467 0.667,0.333,0.967
MnScP	Sc	4.070 12.376	-0.2 0 0.2 0 0 0 0 0 -2.0 -2.0 2.0 2.0	0,0,0.125 0,0,0.375 0,0,0.625 0,0,0.875 0.333,0.667,0.244 0.667,0.333,0.506 0.333,0.667,0.744 0.667,0.333,0.006 0.333,0.667,0.536 0.667,0.333,0.714 0.333,0.667,0.036 0.667,0.333,0.214
MnVP	V	3.891 5.557	-0.1 -0.1 1.0 1.0 0 0	0,0,0.216 0,0,0.716 0.333,0.667,0.062 0.667,0.333,0.562 0.333,0.667,0.472 0.667,0.333,0.972
MnZrSi	Zr	4.197 12.499	2.3 2.2 0 0 0 0 -2.3 -2.2 -0.3 0 0.3 0.0	0.333,0.667,0.030 0.667,0.333,0.220 0.333,0.667,0.249 0.667,0.333,0.501 0.333,0.667,0.749 0.667,0.333,0.001 0.333,0.667,0.529 0.667,0.333,0.720 0,0,0.125 0,0,0.375 0,0,0.625 0,0,0.875
VScP	Sc	4.242 18.393	0.1 -0.1 0.1 -0.1 0.1 -0.1 0 0 0 0 0 0 -1.9 -1.9 -1.9 1.9 1.9 1.9	0,0,0.086 0,0,0.253 0,0,0.420 0,0,0.586 0,0,0.753 0,0,0.920 0.333,0.667,0.153 0.667,0.333,0.319 0.333,0.667,0.486

				0.667,0.333,0.653 0.333,0.667,0.819 0.667,0.333,0.986 0.667,0.333,0.178 0.667,0.333,0.511 0.667,0.333,0.844 0.333,0.667,0.011 0.333,0.667,0.344 0.333,0.667,0.678
VZrP	Zr	4.288 5.921	0 0 1.2 1.1 0 0	0,0,0.245 0,0,0.745 0.333,0.667,0.032 0.667,0.333,0.532 0.333,0.667,0.473 0.667,0.333,0.973
MnNiP	Mn	7.504 6.029	0 0 0 0 0 0 0 0 -2.8 -2.8 -2.9 -2.9 2.9 2.9 2.8 2.8 0 0 0 0 0 0 0 0	0.166,0.333,0.043 0.333,0.166,0.543 0.166,0.834,0.043 0.334,0.666,0.543 0.666,0.334,0.043 0.834,0.166,0.543 0.666,0.833,0.043 0.833,0.666,0.543 0.001,0.5,0.774 0.5,0.001,0.275 0.499,0.5,0.275 0.5,0.5,0.775 0.999,1,0.275 1,1,0.775 0,0.501,0.275 0.501,0,0.774 0.166,0.333,0.432 0.334,0.167,0.932 0.168,0.832,0.432 0.332,0.668,0.932 0.668,0.332,0.432 0.832,0.168,0.932 0.666,0.833,0.432 0.834,0.667,0.932
FeZrP	Zr	4.344 11.135	0 0 0 0 -1.5 -1.5 1.5 1.5 -0.2 -0.2 0.2 0.2	0,0,1 0,0,0.25 0,0,0.5 0,0,0.75 0.333,0.667,0.876 0.667,0.333,0.624 0.333,0.667,0.376 0.667,0.333,0.124 0.333,0.667,0.128 0.667,0.333,0.372 0.333,0.667,0.628 0.667,0.333,0.872
FeTiP	Ti	4.091 10.712	0 0 0 0 -1.3 -1.3 1.3 1.3 -0.3 -0.3 0.3 0.3	0,0,1 0,0,0.25 0,0,0.5 0,0,0.75 0.333,0.667,0.874 0.667,0.333,0.626 0.333,0.667,0.374 0.667,0.333,0.126 0.333,0.667,0.127 0.667,0.333,0.373 0.333,0.667,0.627 0.667,0.333,0.873
VLiAs	Li	4.449 10.715	-0.1 -0.1 0.1 0.1 -2.3 -2.3 0 0 0 0 2.3 2.3	0.333,0.667,0.125 0.667,0.333,0.375 0.333,0.667,0.625 0.667,0.333,0.875 0.333,0.667,0.875 0.667,0.333,0.625 0,0,0 0,0,0.25 0,0,0.5 0,0,0.75 0.333,0.667,0.375 0.667,0.333,0.125
CrLiGe	Li	4.415 5.247	0 0 3.0 3.0 -0.2 -0.2	0,0,0.25 0,0,0.75 0.333,0.667,1 0.667,0.333,0.5 0.333,0.667,0.5 0.667,0.333,1
FeBeSi	Be	7.854 4.701	0 0 0 0 0 0 0 0 0 0 0 0 0 0 0 0 -1.0 -1.0 -1.0 -1.0 1.0 1.0 1.0 1.0	0.002,0,0 0.002,0,0.5 0.998,0.5,0 0.998,0.5,0.5 0.502,0,1 0.502,0,0.5 0.498,0.5,1 0.498,0.5,0.5 0.114,0.315,0.25 0.386,0.185,0.75 0.201,0.815,0.25 0.299,0.685,0.75 0.614,0.315,0.25 0.886,0.185,0.75 0.701,0.815,0.25 0.799,0.685,0.75 0.374,0.661,0.25 0.714,0.339,0.75 0.626,0.839,0.75 0.874,0.661,0.25 0.214,0.339,0.75 0.286,0.161,0.25 0.126,0.839,0.75 0.786,0.161,0.25
VTiGe	Ti	8.836 5.305	0,0,0,0,0,0,0,0,-1.1,-1.1,1.1,-1.1,0,0,0,0,0,0,0,0,1.1,-1.1,1.1,1.1	0.167,0.333,0.75 0.333,0.167,0.25 0.167,0.833,0.75 0.333,0.667,0.25 0.667,0.333,0.75 0.833,0.167,0.25 0.667,0.833,0.75 0.833,0.667,0.25 0.333,0.667,0.75 0.833,0.167,0.75 0.667,0.833,0.25 0.833,0.667,0.75 0,0,0 0,0,0.5 0,0.5,0 0,0.5,0.5 0.5,0,0 0.5,0,0.5 0.5,0.5,0 0.5,0.5,0.5 0.167,0.333,0.25 0.333,0.167,0.75 0.167,0.833,0.25 0.667,0.333,0.25

Table S.11- Predicted magnetic properties, ground state and critical temperatures of phases in the magneto-structural dataset

Phase	Lattice Param. (Å)	Mag. Mom. (μB/atom)	Crystallographic Coordinates (arb.)
FeZrSb	5.559 7.895 10.262	0 0 0 0 0 0 0 0 -2.1 2.1 2.1 -2.1 -2.1 2.1 2.1 -2.1 0 0 0 0 0 0 0 0	0.75,0.125,0.351 0.75,0.375,0.851 0.25,0.375,0.649 0.25,0.125,0.149 0.75,0.625,0.351 0.75,0.875,0.851 0.25,0.875,0.649 0.25,0.625,0.149 0.5,0.125,0.75 0.5,0.875,0.25 0,0.875,0.25 0.5,0.625,0.75 0,0.125,0.75 0.5,0.375,0.25 0,0.375,0.25 0,0.625,0.75 0.25,0.125,0.441 0.25,0.375,0.941 0.75,0.375,0.559 0.75,0.125,0.059 0.25,0.625,0.441 0.25,0.875,0.941 0.75,0.875,0.559 0.75,0.625,0.059
FeLiGe	7.037 3.137 7.616	0 0 0 0 2.0 2.0 1.9 1.9 -0.1 -0.1 -0.1 -0.1	0.046,0.25,0.842 0.954,0.75,0.158 0.454,0.75,0.342 0.546,0.25,0.658 0.109,0.25,0.427 0.891,0.75,0.573 0.391,0.75,0.927 0.609,0.25,0.073 0.239,0.75,0.637 0.761,0.25,0.363 0.261,0.25,0.137 0.739,0.75,0.863
MnTiGe	12.803 3.572 7.647	0 0 -0.1 -0.1 0 0 0.1 0.1 -2.2 -2.1 -2.1 -2.2 2.1 2.1 2.2 2.2 -0.3 -0.3 -0.1 0.1 0.3 0.3 0.1 -0.1	0.378,0.25,0.372 0.372,0.75,0.872 0.124,0.75,0.627 0.126,0.25,0.127 0.878,0.25,0.372 0.872,0.75,0.872 0.624,0.75,0.627 0.626,0.25,0.127 0.432,0.75,0.565 0.570,0.25,0.435 0.680,0.75,0.935 0.818,0.25,0.065 0.070,0.25,0.435 0.180,0.75,0.935 0.318,0.25,0.065 0.932,0.75,0.565 0.021,0.25,0.820 0.229,0.75,0.320 0.478,0.75,0.181 0.272,0.25,0.681 0.521,0.25,0.820 0.729,0.75,0.320 0.978,0.75,0.181 0.772,0.25,0.681

CrLiP	6.682 6.526 7.132	-0.2 0.2 0.2 0.2 -0.2 -0.2 0.2 -0.2 0 0 0 0 0 0 0 0 0 0 0 0 0 0 0 0	0.117,0.125,0.442 0.383,0.375,0.942 0.883,0.375,0.558 0.883,0.875,0.558 0.617,0.125,0.058 0.117,0.625,0.442 0.383,0.875,0.942 0.617,0.625,0.058 0.034,0.125,0.843 0.466,0.375,0.343 0.966,0.375,0.157 0.534,0.125,0.657 0.034,0.625,0.843 0.466,0.875,0.343 0.966,0.875,0.157 0.534,0.625,0.657 0.767,0.125,0.359 0.733,0.375,0.859 0.233,0.375,0.641 0.267,0.125,0.141 0.767,0.625,0.359 0.733,0.875,0.859 0.233,0.875,0.641 0.267,0.625,0.141
CrLiAs	6.817 3.789 14.855	2.7 2.7 2.7 2.7 0.1 0.1 -0.1 -0.1 0.1 0.1 -0.1 -0.1 -2.7 -2.7 -2.7 -2.7 0 0 0 0 0 0 0 0	0.118,0.25,0.226 0.382,0.75,0.476 0.118,0.25,0.726 0.382,0.75,0.976 0.756,0.25,0.182 0.744,0.75,0.432 0.244,0.75,0.318 0.256,0.25,0.068 0.756,0.25,0.682 0.744,0.75,0.932 0.244,0.75,0.818 0.256,0.25,0.568 0.882,0.75,0.274 0.618,0.25,0.024 0.882,0.75,0.774 0.618,0.25,0.524 0.034,0.25,0.418 0.466,0.75,0.168 0.966,0.75,0.082 0.534,0.25,0.332 0.034,0.25,0.918 0.466,0.75,0.668 0.966,0.75,0.582 0.534,0.25,0.832
VLiSb	7.722 3.448 8.426	0 0 0 0 -1.1 -1.1 0 0 0 0 1.1 1.1	0.741,0.25,0.368 0.759,0.75,0.868 0.259,0.75,0.632 0.241,0.25,0.132 0.905,0.75,0.568 0.595,0.25,0.068 0.061,0.25,0.829 0.439,0.75,0.329 0.939,0.75,0.171 0.561,0.25,0.671 0.095,0.25,0.432 0.405,0.75,0.932
FeNbGe	6.288 3.807 7.237	-0.1 0.1 -0.1 0.1 -1.2 -1.2 0 0 0 0 1.2 1.2	0.024,0.25,0.811 0.476,0.75,0.311 0.976,0.75,0.189 0.524,0.25,0.689 0.358,0.75,0.939 0.642,0.25,0.061 0.765,0.25,0.381 0.735,0.75,0.881 0.235,0.75,0.619 0.265,0.25,0.119 0.142,0.25,0.439 0.858,0.75,0.561
FeLiAs	6.655 7.457 6.967	0 0 0 0 0 0 0 0 -2.0 -2.0 -2.0 -2.0 2.0 2.0 2.0 2.0 0 0 0 0 0 0 0 0	0.772,0.125,0.361 0.728,0.375,0.861 0.228,0.375,0.639 0.272,0.125,0.139 0.772,0.625,0.361 0.728,0.875,0.861 0.228,0.875,0.639 0.272,0.625,0.139 0.378,0.375,0.951 0.622,0.125,0.049 0.378,0.875,0.951 0.622,0.625,0.049 0.122,0.125,0.451 0.878,0.375,0.549 0.122,0.625,0.451 0.878,0.875,0.549 0.023,0.125,0.836 0.477,0.375,0.336 0.977,0.375,0.164 0.523,0.125,0.664 0.023,0.625,0.836 0.477,0.875,0.336 0.977,0.875,0.164 0.523,0.625,0.664
CrTiGe	6.631 3.328 7.830	0 0 0 0 0 0 0 0 0 0 0 0	0.053,0.250,0.833 0.947,0.750,0.167 0.447,0.750,0.333 0.553,0.250,0.667 0.120,0.250,0.440 0.880,0.750,0.560 0.380,0.750,0.940 0.620,0.250,0.060 0.255,0.750,0.639 0.745,0.250,0.361 0.245,0.250,0.139 0.755,0.750,0.861
CrNbP	6.262 3.534 7.378	0 0 0 0 1.4 1.4 1.4 1.4 0 0 0 0	0.031,0.25,0.827 0.969,0.75,0.173 0.469,0.75,0.327 0.531,0.25,0.673 0.143,0.25,0.441 0.857,0.75,0.559 0.357,0.75,0.941 0.643,0.25,0.059 0.230,0.75,0.632 0.770,0.25,0.368 0.270,0.25,0.132 0.730,0.75,0.868
VHfAs	6.768 3.572 8.093	0 0 0 0 0 0 0 0 0 0 0 0	0.378,0.25,0.361 0.372,0.75,0.861 0.122,0.75,0.639 0.128,0.25,0.139 0.878,0.25,0.361 0.872,0.75,0.861 0.622,0.75,0.639 0.628,0.25,0.139 0.564,0.25,0.442 0.686,0.75,0.942 0.936,0.75,0.558 0.814,0.25,0.058 0.022,0.25,0.833 0.228,0.75,0.333 0.478,0.75,0.167 0.272,0.25,0.667 0.522,0.25,0.833 0.728,0.75,0.333 0.978,0.75,0.167 0.772,0.25,0.667 0.064,0.25,0.442 0.186,0.75,0.942 0.436,0.75,0.558 0.314,0.25,0.058
VZrAs	6.807 3.613 8.133	0 0 0 0 0 0 0 0 0 0 0 0	0.756,0.25,0.181 0.744,0.75,0.431 0.244,0.75,0.319 0.256,0.25,0.069 0.756,0.25,0.681 0.744,0.75,0.931 0.244,0.75,0.819 0.256,0.25,0.569 0.125,0.25,0.722 0.375,0.75,0.972 0.875,0.75,0.778 0.625,0.25,0.528 0.044,0.25,0.416 0.456,0.75,0.166 0.956,0.75,0.084 0.544,0.25,0.334 0.044,0.25,0.916 0.456,0.75,0.666 0.956,0.75,0.584 0.544,0.25,0.834 0.125,0.25,0.222 0.375,0.75,0.472 0.875,0.75,0.278 0.625,0.25,0.028
VTiP	6.362 3.346 7.660	0 0 0 0 0 0 0 0 0 0 0 0	0.755,0.25,0.180 0.745,0.75,0.430 0.245,0.75,0.320 0.255,0.25,0.070 0.755,0.25,0.680 0.745,0.75,0.930 0.245,0.75,0.820 0.255,0.25,0.570 0.867,0.75,0.280 0.133,0.25,0.720 0.367,0.75,0.970 0.867,0.75,0.780 0.042,0.25,0.416 0.458,0.75,0.166 0.958,0.75,0.084 0.542,0.25,0.334 0.042,0.25,0.916 0.458,0.75,0.666 0.958,0.75,0.584 0.542,0.25,0.834 0.133,0.25,0.220 0.367,0.75,0.470 0.633,0.25,0.030 0.633,0.25,0.530
MnHfP	6.378 3.600 7.458	-0.1 -0.1 -0.1 -0.1 2.0 2.0 2.0 2.0 0 0 0 0	0.032,0.25,0.825 0.968,0.75,0.175 0.468,0.75,0.325 0.532,0.25,0.675 0.136,0.25,0.440 0.864,0.75,0.560 0.364,0.75,0.940 0.636,0.25,0.060 0.233,0.75,0.628 0.767,0.25,0.372 0.267,0.25,0.128 0.733,0.75,0.872

MnZrP	6.439<br>3.636<br>15.027	2.1 2.1 2.1 2.1 2.1 2.1 2.1 2.1 -0.1 -0.1 -0.1 -0.1 -0.1 -0.1 -0.1 -0.1 0 0 0 0 0 0 0 0	0.135,0.25,0.220 0.365,0.75,0.470 0.135,0.25,0.720 0.365,0.75,0.970<br>0.865,0.75,0.280 0.635,0.25,0.030 0.865,0.75,0.780 0.635,0.25,0.530<br>0.030,0.25,0.412 0.470,0.75,0.162 0.970,0.75,0.088 0.530,0.25,0.338<br>0.030,0.25,0.912 0.470,0.75,0.662 0.970,0.75,0.588 0.530,0.25,0.838<br>0.768,0.25,0.187 0.732,0.75,0.437 0.232,0.75,0.313 0.268,0.25,0.063<br>0.768,0.25,0.687 0.732,0.75,0.937 0.232,0.75,0.813 0.268,0.25,0.563
MnTiP	6.150<br>3.473<br>7.202	0 0 0 0 1.8 1.8 1.8 1.8 0 0 0 0	0.262,0.25,0.868 0.738,0.75,0.132 0.238,0.75,0.368 0.762,0.25,0.632<br>0.139,0.25,0.560 0.861,0.75,0.440 0.361,0.75,0.060 0.639,0.25,0.940<br>0.466,0.75,0.672 0.534,0.25,0.328 0.034,0.25,0.172 0.966,0.75,0.828
MnHfSi	13.027<br>3.660<br>7.592	0 0 0 0 0 0 0 0 2.0 1.9 1.9 2.0 0.1 0.1 0 0 -0.1 -0.1 0 0 -1.9 -1.9 -2.0 -2.0	0.387,0.25,0.377 0.363,0.75,0.877 0.114,0.75,0.623 0.136,0.25,0.123<br>0.887,0.25,0.377 0.863,0.75,0.877 0.614,0.75,0.623 0.636,0.25,0.123<br>0.429,0.75,0.565 0.572,0.25,0.435 0.678,0.75,0.935 0.821,0.25,0.065<br>0.014,0.25,0.821 0.236,0.75,0.321 0.485,0.75,0.180 0.265,0.25,0.680<br>0.514,0.25,0.821 0.736,0.75,0.321 0.985,0.75,0.180 0.765,0.25,0.680<br>0.072,0.25,0.435 0.178,0.75,0.935 0.321,0.25,0.065 0.929,0.75,0.565
VNbGe	6.656<br>3.461<br>8.076	0 0 0 0 0 0 0 0 0 0 0 0	0.050,0.250,0.831 0.950,0.750,0.169 0.450,0.750,0.331<br>0.550,0.250,0.669 0.128,0.250,0.439 0.872,0.750,0.561<br>0.372,0.750,0.939 0.628,0.250,0.061 0.251,0.750,0.638<br>0.749,0.250,0.362 0.249,0.250,0.138 0.751,0.750,0.862
MnZrGe	6.724<br>3.783<br>15.658	2.7 2.8 2.7 2.8 0 0 0 0 0 0 0 0 -2.8 -2.7 -2.8 -2.7 0.1 -0.1 -0.1 0.1 0.1 -0.1 -0.1 0.1	0.141,0.25,0.218 0.641,0.25,0.032 0.141,0.25,0.718 0.641,0.25,0.532<br>0.769,0.25,0.187 0.731,0.75,0.437 0.231,0.75,0.313 0.269,0.25,0.063<br>0.769,0.25,0.687 0.731,0.75,0.937 0.231,0.75,0.813 0.269,0.25,0.563<br>0.359,0.75,0.468 0.859,0.75,0.282 0.359,0.75,0.968 0.859,0.75,0.782<br>0.027,0.25,0.411 0.473,0.75,0.161 0.973,0.75,0.089 0.527,0.25,0.339<br>0.027,0.25,0.911 0.473,0.75,0.661 0.973,0.75,0.589 0.527,0.25,0.839
FeLiSb	7.305<br>8.027<br>7.346	0 0 0 0 0 0 0 0 -2.3 -2.3 -2.3 -2.3 2.3 2.3 2.3 2.3 0 0 0 0 0 0 0 0 0.0	0.759,0.125,0.364 0.741,0.375,0.864 0.241,0.375,0.636<br>0.259,0.125,0.136 0.759,0.625,0.364 0.741,0.875,0.864<br>0.241,0.875,0.636 0.259,0.625,0.136 0.396,0.375,0.957<br>0.604,0.125,0.043 0.396,0.875,0.957 0.604,0.625,0.043<br>0.104,0.125,0.457 0.896,0.375,0.543 0.104,0.625,0.457<br>0.896,0.875,0.543 0.019,0.125,0.825 0.481,0.375,0.325<br>0.981,0.375,0.175 0.519,0.125,0.675 0.019,0.625,0.825<br>0.481,0.875,0.325 0.981,0.875,0.175 0.519,0.625,0.675
CrHfSi	6.705<br>3.459<br>7.856	0 0 0 0 0 0 0 0 0 0 0 0	0.043,0.25,0.831 0.957,0.75,0.169 0.457,0.75,0.331 0.543,0.25,0.669<br>0.126,0.25,0.440 0.874,0.75,0.560 0.374,0.75,0.940 0.626,0.25,0.060<br>0.240,0.75,0.633 0.760,0.25,0.367 0.260,0.25,0.133 0.740,0.75,0.867
FeHfAs	12.792<br>3.895<br>7.217	0 0 0 0 0 0 0 0 0.6 0.6 0.6 -0.6 0 0 0 0 0 0 0 0 0.6 -0.6 -0.6 -0.6	0.138,0.25,0.884 0.112,0.75,0.384 0.362,0.75,0.116 0.388,0.25,0.616<br>0.638,0.25,0.884 0.612,0.75,0.384 0.862,0.75,0.116 0.888,0.25,0.616<br>0.425,0.75,0.437 0.575,0.25,0.563 0.925,0.75,0.437 0.825,0.25,0.937<br>0.258,0.25,0.312 0.492,0.75,0.812 0.242,0.75,0.688 0.008,0.25,0.188<br>0.758,0.25,0.312 0.992,0.75,0.812 0.742,0.75,0.688 0.508,0.25,0.188<br>0.075,0.25,0.563 0.175,0.75,0.063 0.325,0.25,0.937 0.675,0.75,0.063
CoCrP	5.694<br>3.519<br>6.716	1.8 1.8 -1.8 -1.8 0.2 -0.2 0.2 -0.2 0 0 0 0	0.026,0.25,0.832 0.974,0.75,0.168 0.474,0.75,0.332 0.526,0.25,0.668<br>0.145,0.25,0.433 0.355,0.75,0.933 0.855,0.75,0.567 0.645,0.25,0.067<br>0.763,0.25,0.376 0.737,0.75,0.876 0.237,0.75,0.624 0.263,0.25,0.124
CoFeP	5.697<br>3.516<br>6.513	1.8 1.8 1.8 1.8 0.3 0.3 0.3 0.3 0 0 0 0	0.032,0.25,0.837 0.968,0.75,0.163 0.468,0.75,0.337 0.532,0.25,0.663<br>0.140,0.25,0.433 0.860,0.75,0.567 0.360,0.75,0.933 0.640,0.25,0.067<br>0.243,0.75,0.620 0.757,0.25,0.380 0.257,0.25,0.120 0.743,0.75,0.880
CrFeP	5.808<br>3.555<br>6.563	2.0 2.0 -2.0 -2.0 0 0 0 0 0 0 0 0	0.025,0.25,0.827 0.475,0.75,0.327 0.975,0.75,0.173 0.525,0.25,0.673<br>0.144,0.25,0.438 0.356,0.75,0.938 0.856,0.75,0.562 0.644,0.25,0.062<br>0.770,0.25,0.376 0.730,0.75,0.876 0.230,0.75,0.624 0.270,0.25,0.124
CrMnP	5.704<br>3.608<br>6.734	-1.8 1.8 -1.8 1.8 -1.0 -1.0 1.0 1.0 0 0 0 0.0	0.026,0.25,0.826 0.474,0.75,0.326 0.974,0.75,0.174 0.526,0.25,0.674<br>0.355,0.75,0.944 0.645,0.25,0.056 0.145,0.25,0.444 0.855,0.75,0.556<br>0.760,0.25,0.372 0.740,0.75,0.872 0.240,0.75,0.628 0.260,0.25,0.128
CrNiP	5.713<br>3.523	0 0 0 0 -2.0 -2.0 0 0 0 0 2.0 2.0	0.142,0.25,0.435 0.358,0.75,0.935 0.858,0.75,0.565 0.642,0.25,0.065<br>0.472,0.75,0.330 0.528,0.25,0.670 0.756,0.25,0.374 0.744,0.75,0.874

	6.848		0.244,0.75,0.626 0.256,0.25,0.126 0.028,0.25,0.830 0.972,0.75,0.170
CrTiSi	6.553<br>3.247<br>7.692	0 0 0 0 0 0 0 0 0 0 0 0	0.048,0.250,0.834 0.952,0.750,0.166 0.452,0.750,0.334<br>0.548,0.250,0.666 0.125,0.250,0.438 0.875,0.750,0.562<br>0.375,0.750,0.938 0.625,0.250,0.062 0.248,0.750,0.637<br>0.752,0.250,0.363 0.252,0.250,0.137 0.748,0.750,0.863
FeZrGe	6.506<br>4.091<br>7.065	0 0 0 0 0 0 0 0 0 0 0 0	0.993,0.250,0.796 0.007,0.750,0.204 0.507,0.750,0.296<br>0.493,0.250,0.704 0.155,0.250,0.437 0.845,0.750,0.563<br>0.345,0.750,0.937 0.655,0.250,0.063 0.216,0.750,0.605<br>0.784,0.250,0.395 0.284,0.250,0.105 0.716,0.750,0.895
FeMnP	5.863<br>3.521<br>13.268	0 0 0 0 0 0 0 0 -2.9 -2.9 -2.8 -2.8 0.4 -0.4 0.4 0.4 -0.4 0.4 -0.4 -0.4 2.9 2.8 2.8 2.9	0.778,0.25,0.186 0.723,0.75,0.437 0.223,0.75,0.313 0.278,0.25,0.064<br>0.778,0.25,0.686 0.723,0.75,0.937 0.223,0.75,0.813 0.278,0.25,0.564<br>0.523,0.25,0.333 0.023,0.25,0.917 0.476,0.75,0.667 0.976,0.75,0.583<br>0.151,0.25,0.218 0.349,0.75,0.467 0.849,0.75,0.283 0.651,0.25,0.032<br>0.151,0.25,0.718 0.349,0.75,0.967 0.849,0.75,0.783 0.651,0.25,0.532<br>0.023,0.25,0.417 0.476,0.75,0.167 0.976,0.75,0.083 0.523,0.25,0.833
FeNbSi	6.216<br>3.733<br>6.987	0 0 0 0 0 0 0 0 0 0 0 0	0.014,0.250,0.812 0.986,0.750,0.188 0.486,0.750,0.312<br>0.514,0.250,0.688 0.147,0.250,0.434 0.853,0.750,0.566<br>0.353,0.750,0.934 0.647,0.250,0.066 0.221,0.750,0.613<br>0.779,0.250,0.387 0.279,0.250,0.113 0.721,0.750,0.887
FeScP	6.236<br>3.814<br>6.922	0 0 0 0 0 0 0 0 0 0 0 0	0.007,0.250,0.812 0.993,0.750,0.188 0.493,0.750,0.312<br>0.507,0.250,0.688 0.151,0.250,0.439 0.849,0.750,0.561<br>0.349,0.750,0.939 0.651,0.250,0.061 0.210,0.750,0.614<br>0.790,0.250,0.386 0.290,0.250,0.114 0.710,0.750,0.886
FeZrSi	6.452<br>4.000<br>6.927	0 0 0 0 0 0 0 0 0 0 0 0	0.990,0.250,0.803 0.010,0.750,0.197 0.510,0.750,0.303<br>0.490,0.250,0.697 0.154,0.250,0.436 0.846,0.750,0.564<br>0.346,0.750,0.936 0.654,0.250,0.064 0.207,0.750,0.605<br>0.793,0.250,0.395 0.293,0.250,0.105 0.707,0.750,0.895
MnNbP	12.415<br>3.525<br>7.260	0 0 0 0 0 0 0 0 0 0 0 0 0 0 0 0 -1.6 -1.6 -1.6 -1.6 1.6 1.6 1.6 1.6	0.386,0.25,0.370 0.364,0.75,0.870 0.114,0.75,0.630 0.136,0.25,0.130<br>0.886,0.25,0.370 0.864,0.75,0.870 0.614,0.75,0.630 0.636,0.25,0.130<br>0.015,0.25,0.829 0.235,0.75,0.329 0.485,0.75,0.171 0.265,0.25,0.671<br>0.515,0.25,0.829 0.735,0.75,0.329 0.985,0.75,0.171 0.765,0.25,0.671<br>0.179,0.75,0.939 0.321,0.25,0.061 0.679,0.75,0.939 0.821,0.25,0.061<br>0.071,0.25,0.439 0.429,0.75,0.561 0.570,0.25,0.439 0.929,0.75,0.561
MnScP	12.819<br>3.640<br>7.424	-0.1 -0.1 0 0 0.1 0.1 0 0 0 0 0 0 0 0 0 0 -2.0 -1.9 -1.9 -2.0 1.9 1.9 2.0 2.0	0.015,0.25,0.825 0.235,0.75,0.325 0.484,0.75,0.175 0.266,0.25,0.675<br>0.515,0.25,0.825 0.735,0.75,0.325 0.984,0.75,0.175 0.766,0.25,0.675<br>0.387,0.25,0.371 0.363,0.75,0.871 0.114,0.75,0.627 0.136,0.25,0.127<br>0.887,0.25,0.371 0.863,0.75,0.871 0.614,0.75,0.627 0.636,0.25,0.127<br>0.431,0.75,0.560 0.570,0.25,0.441 0.680,0.75,0.941 0.819,0.25,0.060<br>0.070,0.25,0.441 0.180,0.75,0.941 0.319,0.25,0.060 0.931,0.75,0.560
MnVP	5.966<br>3.407<br>7.016	0 0 0 0 -1.5 -1.5 1.5 1.5 -0.2 0.2 -0.2 0.2	0.763,0.25,0.366 0.737,0.75,0.866 0.237,0.75,0.634 0.263,0.25,0.134<br>0.357,0.75,0.940 0.643,0.25,0.060 0.143,0.25,0.440 0.857,0.75,0.560<br>0.033,0.25,0.831 0.467,0.75,0.331 0.967,0.75,0.169 0.533,0.25,0.669
MnZrSi	6.516<br>3.821<br>15.188	2.4 2.4 2.4 2.4 0 0 0 0 0 0 0 0 -2.4 -2.4 -2.4 -2.4 0.1 -0.1 -0.1 0.1 0.1 -0.1 -0.1 0.1	0.147,0.25,0.218 0.647,0.25,0.032 0.147,0.25,0.718 0.647,0.25,0.532<br>0.776,0.25,0.189 0.724,0.75,0.439 0.224,0.75,0.311 0.276,0.25,0.061<br>0.776,0.25,0.689 0.724,0.75,0.939 0.224,0.75,0.811 0.276,0.25,0.561<br>0.353,0.75,0.468 0.853,0.75,0.282 0.353,0.75,0.968 0.853,0.75,0.782<br>0.021,0.25,0.410 0.479,0.75,0.160 0.979,0.75,0.090 0.521,0.25,0.340<br>0.021,0.25,0.910 0.479,0.75,0.660 0.979,0.75,0.590 0.521,0.25,0.840
VScP	6.640<br>3.513<br>7.856	0 0 0 0 0 0 0 0 0 0 0 0	0.043,0.25,0.835 0.957,0.75,0.165 0.457,0.75,0.335 0.543,0.25,0.665<br>0.125,0.25,0.447 0.875,0.75,0.553 0.375,0.75,0.947 0.625,0.25,0.053<br>0.239,0.75,0.639 0.761,0.25,0.361 0.261,0.25,0.139 0.739,0.75,0.861
VZrP	6.628<br>3.516<br>7.908	0 0 0 0 0 0 0 0 0 0 0 0	0.039,0.25,0.830 0.961,0.75,0.170 0.461,0.75,0.330 0.539,0.25,0.670<br>0.129,0.25,0.442 0.871,0.75,0.558 0.371,0.75,0.942 0.629,0.25,0.058<br>0.238,0.75,0.637 0.762,0.25,0.363 0.262,0.25,0.137 0.738,0.75,0.863
MnNiP	5.856<br>3.439	2.3 2.3 2.3 2.3 -0.1 -0.1 -0.1 -0.1 -0.1 -0.1 -0.1 -0.1	0.032,0.25,0.841 0.968,0.75,0.159 0.468,0.75,0.341 0.532,0.25,0.659<br>0.139,0.25,0.434 0.861,0.75,0.566 0.361,0.75,0.934 0.639,0.25,0.066

	6.749		0.241,0.75,0.629 0.759,0.25,0.371 0.259,0.25,0.129 0.741,0.75,0.871
FeZrP	6.318<br>3.757<br>7.064	0 0 0 0 0 0 0 0 0 0 0 0	0.283,0.25,0.444 0.217,0.75,0.194 0.717,0.75,0.056 0.783,0.25,0.306<br>0.283,0.25,0.944 0.217,0.75,0.694 0.717,0.75,0.556 0.783,0.25,0.806<br>0.646,0.25,0.469 0.146,0.25,0.781 0.354,0.75,0.531 0.854,0.75,0.719<br>0.146,0.25,0.281 0.354,0.75,0.031 0.854,0.75,0.219 0.646,0.25,0.969<br>0.514,0.25,0.157 0.986,0.75,0.407 0.486,0.75,0.343 0.014,0.25,0.093<br>0.514,0.25,0.657 0.986,0.75,0.907 0.486,0.75,0.843 0.014,0.25,0.593
FeTiP	5.994<br>3.616<br>6.791	0 0 0 0 0 0 0 0 0 0 0 0	0.276,0.25,0.440 0.224,0.75,0.190 0.724,0.75,0.060 0.776,0.25,0.310<br>0.276,0.25,0.940 0.224,0.75,0.690 0.724,0.75,0.560 0.776,0.25,0.810<br>0.351,0.75,0.031 0.649,0.25,0.469 0.351,0.75,0.531 0.649,0.25,0.969<br>0.149,0.25,0.281 0.851,0.75,0.219 0.149,0.25,0.781 0.851,0.75,0.719<br>0.521,0.25,0.159 0.979,0.75,0.409 0.479,0.75,0.341 0.021,0.25,0.091<br>0.521,0.25,0.659 0.979,0.75,0.909 0.479,0.75,0.841 0.021,0.25,0.591
VLiAs	6.954<br>3.510<br>7.705	0 0 0 0 0 0 0 0 0 0 0 0	0.375,0.25,0.363 0.375,0.75,0.863 0.125,0.75,0.637 0.125,0.25,0.137<br>0.875,0.25,0.363 0.875,0.75,0.863 0.625,0.75,0.637 0.625,0.25,0.137<br>0.557,0.25,0.444 0.693,0.75,0.944 0.943,0.75,0.556 0.807,0.25,0.056<br>0.020,0.25,0.835 0.230,0.75,0.335 0.480,0.75,0.165 0.270,0.25,0.665<br>0.520,0.25,0.835 0.730,0.75,0.335 0.980,0.75,0.165 0.770,0.25,0.665<br>0.057,0.25,0.444 0.193,0.75,0.944 0.443,0.75,0.556 0.307,0.25,0.056
CrLiGe	14.549<br>3.027<br>7.972	1.7 1.7 1.7 1.7 0.1 0.1 -0.1 -0.1 0.1 0.1 -0.1 -0.1 -1.7 -1.7 -1.7 -1.7 0 0 0 0 0 0 0 0.0	0.050,0.25,0.432 0.200,0.75,0.932 0.550,0.25,0.432 0.700,0.75,0.932<br>0.376,0.25,0.354 0.374,0.75,0.854 0.124,0.75,0.646 0.126,0.25,0.146<br>0.876,0.25,0.354 0.874,0.75,0.854 0.624,0.75,0.646 0.626,0.25,0.146<br>0.450,0.75,0.568 0.300,0.25,0.068 0.950,0.75,0.568 0.800,0.25,0.068<br>0.027,0.25,0.847 0.223,0.75,0.347 0.473,0.75,0.153 0.277,0.25,0.653<br>0.527,0.25,0.847 0.723,0.75,0.347 0.973,0.75,0.153 0.777,0.25,0.653
FeBeSi	4.039<br>3.939<br>7.460	0 0 0 0 0 0 0 0 0 0 0 0	0.25,0.25,0.416 0.25,0.75,0.166 0.75,0.75,0.084 0.75,0.25,0.334<br>0.25,0.25,0.916 0.25,0.75,0.666 0.75,0.75,0.584 0.75,0.25,0.834<br>0.75,0.25,0.171 0.75,0.75,0.421 0.25,0.75,0.329 0.25,0.25,0.079<br>0.75,0.25,0.671 0.75,0.75,0.921 0.25,0.75,0.829 0.25,0.25,0.579<br>0.25,0.25,0.748 0.25,0.75,0.998 0.75,0.75,0.752 0.75,0.25,0.502<br>0.25,0.25,0.248 0.25,0.75,0.498 0.75,0.75,0.252 0.75,0.25,0.002
VTiGe	6.661<br>3.390<br>8.065	0 0 0 0 0 0 0 0 -2.1 2.1 2.1 -2.1 -2.1 2.1 2.1 -2.1 0 0 0 0 0 0 0 0	0.743,0.25,0.180 0.757,0.75,0.430 0.257,0.75,0.320 0.243,0.25,0.070<br>0.743,0.25,0.680 0.757,0.75,0.930 0.257,0.75,0.820 0.243,0.25,0.570<br>0.122,0.25,0.720 0.378,0.75,0.970 0.878,0.75,0.780 0.622,0.25,0.530<br>0.057,0.25,0.417 0.443,0.75,0.167 0.943,0.75,0.083 0.557,0.25,0.333<br>0.057,0.25,0.917 0.443,0.75,0.667 0.943,0.75,0.583 0.557,0.25,0.833<br>0.122,0.25,0.220 0.378,0.75,0.470 0.878,0.75,0.280 0.622,0.25,0.030

Table S.12- Experimental and theoretical lattice parameters for the parent compounds with structural phase transition, along with the $\Delta E_0/K_b$ in the case of DFT and the experimental T_m for comparison.

Phase	Orthorhombic					Hexagonal				
	**a (Å)**	**b (Å)**	**c (Å)**	**Magnetic State**	**Mag. Mom. (μB/atom)**	**a (Å)**	**c (Å)**	**Magnetic State**	**Mn/Fe Mag. Mom. (μB/atom)**	**ΔE_0 / T_m^{exp} (K)**
CoMnSi	5.715	3.651	6.862	FM	2.94 Mn<br>0.66 Co	3.964	4.989	AFM 122	2.50 Mn	425
CoMnSi (Exp.)[a,b,c,d]	5.864	3.687	6.855	AFM/FM*	2.6 Mn<br>0.4 Co	4.03 (1000ºC)	5.29	---	---	1190
CoMnGe	5.825	3.780	7.085	FM	3.18 Mn<br>0.67 Co	4.083	5.133	FM	2.73 Mn<br>0.45 Co	483
CoMnGe (Exp.)[a,e,f]	5.957<br>5.986	3.817<br>3.824	7.054<br>7.073	FM	3.16 Mn<br>0.89 Co	4.10<br>4.070	5.36<br>5.292	---	---	398-458
MnNiGe	6.015	3.660	7.097	AFM 111	3.08 Mn	4.08	5.252	AFM 113	2.99 Mn	349
MnNiGe (Exp.)[a,g]	6.042	3.755	7.086	AFM	2.86 Mn	3.822	5.952	AFM	---	470-493

MnNiSi	5.834	3.557	6.893	FM	2.76 Mn	3.947	5.125	FM	2.45 Mn	693
MnNiSi (Exp.)[a,g]	5.897	3.612	6.916	FM	2.70 Mn	4.04 (1000ºC)	5.38	---	---	1206
FeNiSi (FM)	5.466	3.623	6.857	FM	1.74 Fe	3.913	4.974	AFM 221	1.85 Fe	437
FeNiSi (NM)	4.987	3.686	7.075	NM	---	---	---	---	---	---
FeNiSi (Exp.)[h]	5.007	3.753	7.149	FM	0.96 Fe	---	---	---	---	1164

*- metamagnetic transition to FM at higher temperatures
a-10.1021/ic50147a032
b-10.1103/PhysRevB.74.224436
c-10.1002/pssa.2210450231
d-10.1016/0304-8853(89)90188-1
e-10.1016/0304-8853(82)90087-7
f-10.3379/jmsjmag.23.418
g-10.1002/pssa.2210640140
h-10.1021/ic980223e

Table S.13- Values of COHP for the DFT and experimental FeNiSi lattice parameters, showing both the nearest-neighbour bonds and sum up to a cut-off of 4.5 Å, for spin up and down channels. The more negative values imply greater stability.

	Orthorhombic					Hexagonal		
**Bond**	**-ICOHP 1**	**Dist. 1**	**-ICOHP 2**	**Dist. 2**	**Sum Up/Down**	**-ICOHP 1**	**Dist. NN**	**Sum Up/Down**
**FeNiSi FM DFT lattice**								
Fe-Fe	0.11/0.25	2.87	0.03/0.09	3.13	0.31/0.71	0.21/0.49	2.47	0.49/1.05
Fe-Ni	0.13/0.20	2.64	0.11/0.18	2.74	0.84/1.28	0.15/0.23	2.59	0.95/1.47
Fe-Si	0.74/0.85	2.38	0.53/0.60	2.56	3.41/3.86	0.48/0.58	2.59	3.02/3.58
Ni-Si	0.84/0.81	2.25	0.86/0.82	2.28	3.51/3.35	0.83/0.79	2.28	3.53/3.29
Ni-Ni	0.15/0.15	2.57	0.01/0.02	3.80	0.48/0.49	0.01/0.00	3.36	0.14/0.12
Si-Si	0.24/0.20	3.23	0.13/0.10	3.60	1.35/1.14	0.19/0.16	3.36	1.54/1.34
**FeNiSi experimental lattice**								
Fe-Fe	0.22/0.42	2.55	0.02/0.03	3.75	0.51/0.93	---	---	---
Fe-Ni	0.14/0.22	2.59	0.13/0.20	2.70	0.84/1.29	---	---	---
Fe-Si	0.70/0.80	2.39	0.61/0.69	2.47	3.40/3.82	---	---	---
Ni-Si	0.80/0.77	2.31	0.69/0.68	2.36	3.39/3.23	---	---	---
Ni-Ni	0.1/0.1	2.71	0.01/0.01	3.87	0.36/0.38	---	---	---
Si-Si	0.26/0.23	3.20	0.26/0.23	3.25	1.57/1.38	---	---	---

Table S.14- Prediction energy and Curie temperature (CTW) difference between phases and respective predicted structural transition temperature (T_m) along with maximum magnetization difference between phases.

**#**	**Phase**	**$E^{ort.}$-$E^{hex.}$ (eV/atom)**	**T_m (K)**	**CTW (K)**	**ΔM (μB/atom)**
1	FeZrSb	-0.002	140	0	0.00
2	FeLiGe	-0.031	500	0	0.60
3	MnTiGe	-0.094	540	200	0.71
4	CrLiP	-0.141	830	0	0.00
5	CrLiAs	-0.093	850	0	0.00
6	VLiSb	-0.109	870	0	0.99
7	FeNbGe	-0.065	920	45	0.00
8	FeLiAs	-0.130	930	0	0.00

9	CrTiGe	-0.136	1190	750	0.67
10	CrNbP	-0.378	1400	0	0.00
11	VHfAs	-0.465	1400	260	0.57
12	VZrAs	-0.446	1400	395	0.61
13	VTiP	-0.492	1400	65	0.35
14	MnHfP	-0.372	1400	550	0.65
15	MnZrP	-0.318	1400	545	0.66
16	MnTiP	-0.652	1400	420	0.61
17	MnHfSi	-0.237	1400	90	0.76
18	VNbGe	-0.227	1400	155	0.30
19	MnZrGe	-0.172	1400	145	0.95
20	FeLiSb	-0.106	1400	0	0.00
21	CrHfSi	-0.265	1400	565	0.69
22	FeHfAs	-0.713	1400	0	0.07
23	CoCrP	-0.178	1400	0	0.00
24	CoFeP	-0.197	1400	200	0.14
25	CrFeP	-0.300	1400	0	0.00
26	CrMnP	-0.273	1400	0	0.00
27	CrNiP	-0.173	1400	0	1.00
28	CrTiSi	-0.198	1400	585	0.64
29	FeZrGe	-0.115	1400	0	0.00
30	FeMnP	-0.188	1400	300	0.00
31	FeNbSi	-0.145	1400	150	0.30
32	FeScP	-0.243	1400	0	0.00
33	FeZrSi	-0.182	1400	0	0.00
34	MnNbP	-0.312	1400	190	0.63
35	MnScP	-0.301	1400	180	0.00
36	MnVP	-0.233	1400	5	0.92
37	MnZrSi	-0.212	1400	0	0.00
38	VScP	-0.491	1400	0	0.00
39	VZrP	-0.573	1400	0	0.43
40	MnNiP	-0.172	1400	0	0.73
41	FeZrP	-1.004	1400	0	0.00
42	FeTiP	-0.931	1400	0	0.00
43	VLiAs	-0.175	1400	0	0.00
44	CrLiGe	-0.013	1400	600	1.01
45	FeBeSi	-0.110	1400	0	0.00
46	VTiGe	-0.208	1400	0	0.30

Table S.15- Possible paths for isostructural alloying, along with formation energy, distance to the convex hull, magnetic moment and space group number in brackets.

**Phase**	**Hex. Form. Energy (eV/atom) Dist. to convex hull (eV/atom) Mag. Mom (µB/atom) SPG nº**
FeZrSb	FeZrMg (-0.001 0.180 0.413 194) FeTiSb (-0.001 0.067 0.315 186) FeZrIn (-0.301 0.193 0.588 194) FeZrSn (-0.482 0.087 0.628 186) FeZrBi (-0.629 0.230 0.327 186) FeHfSb (-0.462 0.040 0.315 186) FeNbSb (-0.462 0.204 0.001 186)
FeLiGe	FeVGe (-0.292 0.033 0.000 194) FeWGe (-0.050 0.143 0.001 186) MnLiGe (-0.073 0.000 1.126 194)
MnTiGe	MnTiGa (-0.285 0.038 0.649 194) MnTiIn (-0.285 0.178 0.648 194) MnCuGe (-0.667 0.097 1.075 194)

CrLiP	CrLiSn (-0.069 0.036 1.063 194) Li_2P (-0.069 0.212 1.230 194) CrLiSb (-0.233 0.000 1.334 194) CrLiBi (-0.233 0.054 1.341 194)
CrLiAs	CrLiSn (-0.069 0.036 1.063 194) Li_2P (-0.069 0.212 1.230 194) CrLiSb (-0.286 0.000 1.334 194) CrLiBi (-0.286 0.054 1.341 194)
VLiSb	VLiSn (-0.163 0.000 0.498 194) CrLiSb (0.007 0.000 1.334 194) CoVSb (-0.202 0.162 0.382 194) NiVSb (-0.379 0.165 0.039 194)
FeNbGe	FeNbSn (-0.469 0.092 0.453 186) FeNbSb (-0.371 0.204 0.001 186) FeNbBi (-0.371 0.336 0.001 186) FeVGe (-0.451 0.033 0.000 194) FeWGe (-0.050 0.143 0.001 186)
FeLiAs	
CrTiGe	CrTiSn (-0.394 0.163 0.644 194) CrTiPb (-0.394 0.317 0.658 194) CrTiBi (-0.109 0.348 0.705 186) CrScTi (-0.296 0.275 0.409 194)
CrNbP	CrNbIn (-0.088 0.224 0.757 186) CrNbSn (-0.142 0.125 0.785 194) CrNbPb (-0.142 0.404 0.797 194) CrNbSb (-0.103 0.273 0.827 186) CrNbBi (-0.103 0.431 0.905 194)
VHfAs	
VZrAs	
VTiP	VTiAl (-0.295 0.043 0.000 194) VTiGa (-0.295 0.001 0.000 194) VHfTi (-0.545 0.131 0.002 194)
MnHfP	MnMnZn (-0.721 0.190 0.823 194) MnHfAl (-0.252 0.093 0.702 194) MnHfIn (-0.296 0.171 0.865 194) MnMnSn (-0.359 0.067 0.967 194) MnMnPb (-0.359 0.265 0.985 194) NiHfP (-0.364 0.000 0.001 194) MnMnZn (-0.097 0.190 0.823 194)
MnZrP	MnZrMg (-0.077 0.218 0.677 194) MnZrZn (-0.041 0.173 0.851 194) MnZrAl (-0.260 0.097 0.724 194) MnZrIn (-0.324 0.139 0.866 194) MnZrSn (-0.419 0.093 0.991 194) MnZrPb (-0.419 0.180 0.994 194) NiZrP (-0.364 0.000 0.000 186) MnZrZn (-0.041 0.173 0.851 194)
MnTiP	MnTiGa (-0.285 0.038 0.649 194) MnTiIn (-0.285 0.178 0.648 194)
MnHfSi	MnMnZn (-0.510 0.190 0.823 194) MnHfAl (-0.252 0.093 0.702 194) MnHfIn (-0.296 0.171 0.865 194) MnMnSn (-0.359 0.067 0.967 194) MnMnPb (-0.359 0.265 0.985 194) MnMnZn (-0.100 0.190 0.823 194)
VNbGe	VVZn (-0.391 0.132 0.000 194) NiVNb (-0.411 0.156 0.000 194) VVZn (-0.056 0.132 0.000 194)
MnZrGe	MnZrAl (-0.260 0.097 0.724 194) MnZrIn (-0.324 0.139 0.866 194) MnZrSn (-0.492 0.093 0.991 194) MnZrPb (-0.492 0.180 0.994 194) MnLiGe (-0.017 0.000 1.126 194) MnCuGe (-0.787 0.097 1.075 194)
FeLiSb	FeHfSb (-0.427 0.040 0.315 186) FeNbSb (-0.427 0.204 0.001 186) CrLiSb (0.013 0.000 1.334 194)
CrHfSi	CrHfIn (-0.134 0.245 0.901 194) CrHfSn (-0.241 0.170 0.754 194) CrHfPb (-0.241 0.307 0.890 194) CrHfBi (-0.099 0.351 0.943 194) CrBeSi (-0.022 0.137 0.005 194)
FeHfAs	FeHfSb (-0.839 0.040 0.315 186) FeHfBi (-0.839 0.292 0.321 186)
CoCrP	CoCrGe (-0.306 0.072 0.799 194) CoCrSb (-0.085 0.223 0.869 194) CoYP (-0.524 0.015 0.045 194)
CoFeP	CoYP (-0.878 0.015 0.045 194)
CrFeP	
CrMnP	
CrNiP	CrNiGa (-0.184 0.164 0.790 194) NiNiSn (-0.132 0.192 1.140 194) NiYP (-0.529 0.000 0.001 186) NiZrP (-0.524 0.000 0.000 186) NiHfP (-0.504 0.000 0.001 194)
CrTiSi	CrTiSn (-0.285 0.163 0.644 194) CrTiPb (-0.285 0.317 0.658 194) CrTiBi (-0.109 0.348 0.705 186) CrScTi (-0.321 0.275 0.409 194) CrCuTi (-0.030 0.267 0.371 194)
FeZrGe	FeZrMg (-0.007 0.180 0.413 194) FeZrIn (-0.301 0.193 0.588 194) FeZrSn (-0.594 0.087 0.628 186) FeZrBi (-0.427 0.230 0.327 186) FeVGe (-0.292 0.033 0.000 194) FeWGe (-0.050 0.143 0.001 186)
FeMnP	

FeNbSi	FeNbMg (-0.023 0.233 0.555 194) FeNbSn (-0.292 0.092 0.453 186) FeNbSb (-0.371 0.204 0.001 186) FeNbBi (-0.371 0.336 0.001 186)
FeScP	FeScIn (-0.309 0.225 0.665 194)
FeZrSi	FeZrMg (-0.049 0.180 0.413 194) FeZrIn (-0.301 0.193 0.588 194) FeZrSn (-0.482 0.087 0.628 186) FeZrBi (-0.427 0.230 0.327 186)
MnNbP	MnNbZn (-0.721 0.158 0.777 194) MnNbAl (-0.174 0.098 0.716 194) MnNbGa (-0.174 0.037 0.754 194) MnNbIn (-0.174 0.160 0.814 186) MnNbSb (-0.275 0.175 0.430 186) MnNbZn (-0.097 0.158 0.777 194)
MnScP	MnScZn (-0.041 0.188 1.152 194) MnScAl (-0.112 0.076 0.883 194) MnScGa (-0.112 0.092 0.963 194) MnScIn (-0.112 0.155 1.030 194) MnScSn (-0.442 0.081 0.891 194) MnScPb (-0.442 0.213 0.923 194) MnScZn (-0.041 0.188 1.152 194)
MnVP	MnVGa (-0.190 0.078 0.574 194)
MnZrSi	MnZrAl (-0.260 0.097 0.724 194) MnZrIn (-0.324 0.139 0.866 194) MnZrSn (-0.419 0.093 0.991 194) MnZrPb (-0.419 0.180 0.994 194) MnLiSi (-0.017 0.121 0.884 194)
VScP	
VZrP	NiZrP (-0.065 0.000 0.000 186)
MnNiP	MnNiAl (-0.081 0.102 1.064 194) MnNiSn (-0.228 0.080 1.154 194) NiYP (-0.703 0.000 0.001 186) NiZrP (-0.732 0.000 0.000 186) NiHfP (-0.726 0.000 0.001 194)
FeZrP	FeZrMg (-0.110 0.180 0.413 194) FeZrIn (-0.301 0.193 0.588 194) FeZrSn (-0.482 0.087 0.628 186) FeZrBi (-0.427 0.230 0.327 186) NiZrP (-0.378 0.000 0.000 186)
FeTiP	FeTiSb (-0.571 0.067 0.315 186)
VLiAs	VLiSn (-0.163 0.000 0.498 194)
CrLiGe	CrLiSn (-0.022 0.036 1.063 194) CrLiSb (-0.233 0.000 1.334 194) CrLiBi (-0.233 0.054 1.341 194) CrBeGe (-0.233 0.214 0.098 194) CoCrGe (-0.120 0.072 0.799 194)
FeBeSi	CrBeSi (-0.207 0.137 0.005 194)
VTiGe	VTiAl (-0.295 0.043 0.000 194) VTiGa (-0.295 0.001 0.000 194) VHfTi (-0.311 0.131 0.002 194) FeVGe (-0.013 0.033 0.000 194)

Table S.16- Possible paths for isostructural alloying for known MM’X parent phases, along with formation energy, distance to the convex hull, magnetic moment and space group number in brackets.

**Phase**	**Hex. Form. Energy (eV/atom) Dist. to convex hull (eV/atom) Mag. Mom (μB/atom) SPG nº**
MnNiGe	**MnNiAl**[c] (-0.081 0.102 1.06 194) **MnNiSn**[d] (-0.431 0.080 1.153 194) MnLiGe (0.234 0 1.126 194) **FeNiGe**[a,b] (-0.073 0.065 0.788 186) MnCuGe (-0.190 0.097 1.075 194) NiCuGe (-0.190 0.072 0.000 194) MnNiTi (-0.185 0.183 0.690 194) MnNiGa (-0.254 0.013 1.088 194) MnNiIn (0.017 0.144 1.261 194) MnNiSb (-0.036 0.170 1.133 194) NiHfGe (-0.745 0.000 0.0000 194) CrNiGe (-0.132 0.098 0.958 194)
CoMnGe	CoMnSn (-0.414 0.094 1.23 194) CoMnSb (-0.172 0.143 1.173 194) MnLiGe (-0.224 0 1.126 194) **CoCrGe**[e] (-0.046 0.072 0.799 194) **CoCuGe**[f] (-0.228 0.106 0.006 186) MnCuGe (-0.228 0.097 1.07 194) CoFeGe (-0.109 0.047 0.904 194)
MnNiSi	MnNiAl (-0.081 0.102 1.064 194) MnNiSn (-0.228 0.080 1.154 194) MnLiSi (-0.420 0.121 0.884 194) NiCuSi (-0.414 0.046 0.000 194) MnNiTi (-0.185 0.183 0.690 194) MnNiGa (-0.254 0.013 1.088 194) MnNiIn (0.017 0.144 1.261 194) MnNiSb (-0.036 0.170 1.132 194)
CoMnSi	CoMnSn (-0.183 0.094 1.232 194) CoMnSb (-0.172 0.143 1.173 194)
FeNiSi	FeNiGa (-0.313 0.078 0.873 194) **FeNiGe**[a] (-0.384 0.065 0.789 194) FeNiSn (-0.003 0.161 194)

a- 10.1088/1361-6463/aa8e89
b- 10.1038/ncomms1868

c-10.1063/1.3681798
d-10.1109/TMAG.2011.2159964
e-10.1063/1.3399774
f-10.1103/PhysRevApplied.13.054003

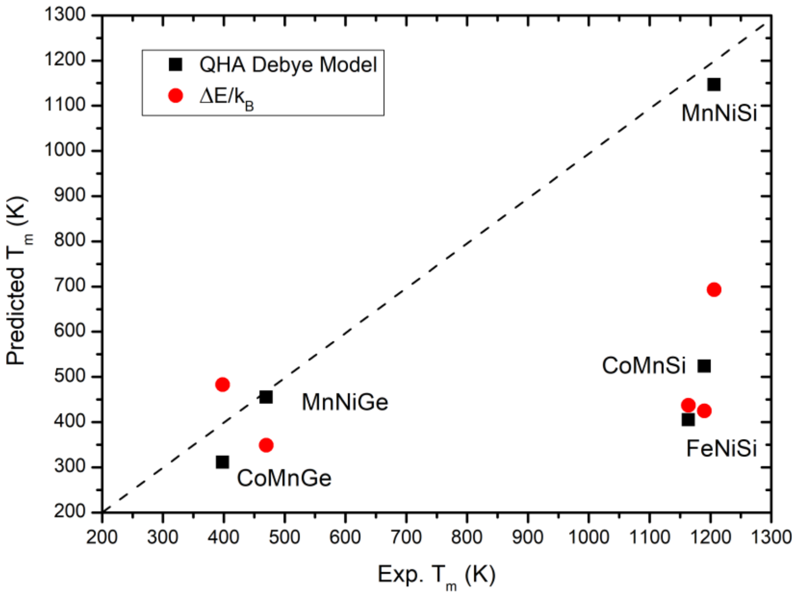

Figure S. 3: Comparison of predicted and experimental transition temperatures for known stoichiometric MM'X, for the QHA model and using the energy difference between martensite and austenite ($\Delta E/K_B$).

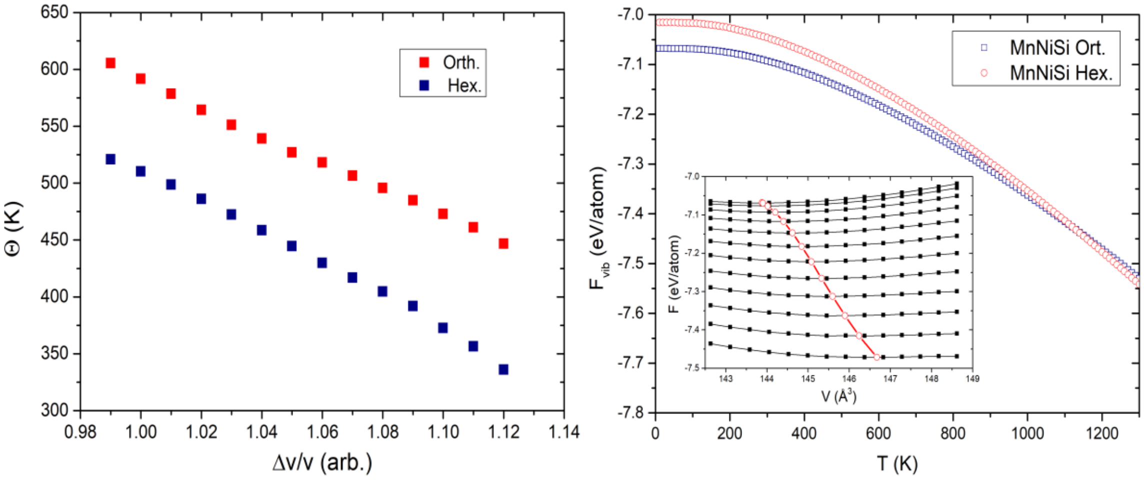

Figure S. 4: QHA Debye Model of MnNiSi, on the left the Debye temperature as function of volume. On the right, the corresponding free energies from the QHA Debye model, with inset showing the minimization procedure at each temperature.

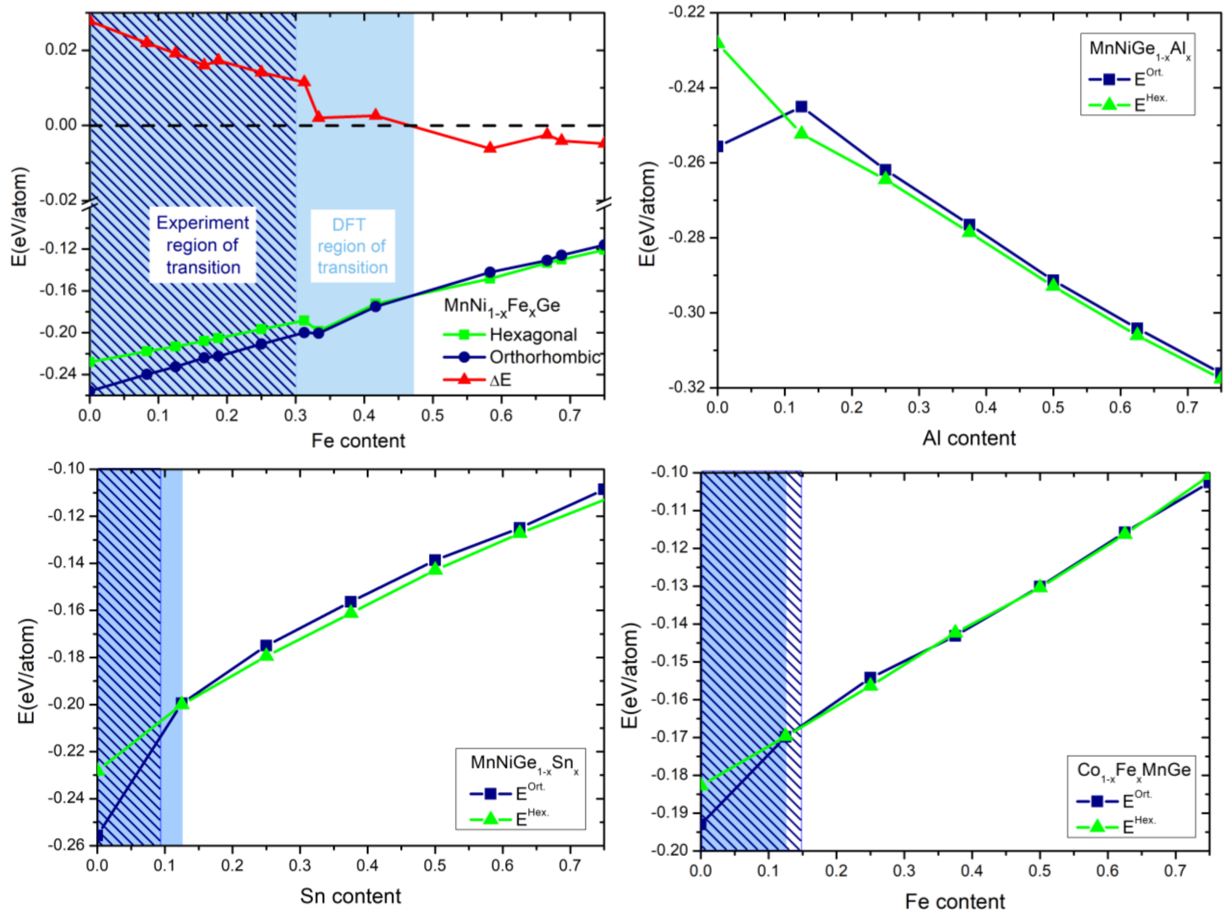

Figure S. 5: Energies for the orthorhombic and hexagonal phases Mn-Fe-NiGe SQS's (top left), MnNiGe-Al (top right), MnNiGe-Sn (bottom left) and Co-FeMnGe. Stripes represent the compositional region where a transition occurs from experiment (i.e. orthorhombic ground state) and in light blue the indication of allowed transition from DFT energy between both phases. Note that the coincidence between points indicate that the orthorhombic phase is no longer stable and relaxes to the hexagonal phase. Respective references for experimental substitutions: 10.1038/ncomms1868, 10.1063/1.3681798, 10.1109/TMAG.2011.2159964, 10.1109/TMAG.2006.884516.

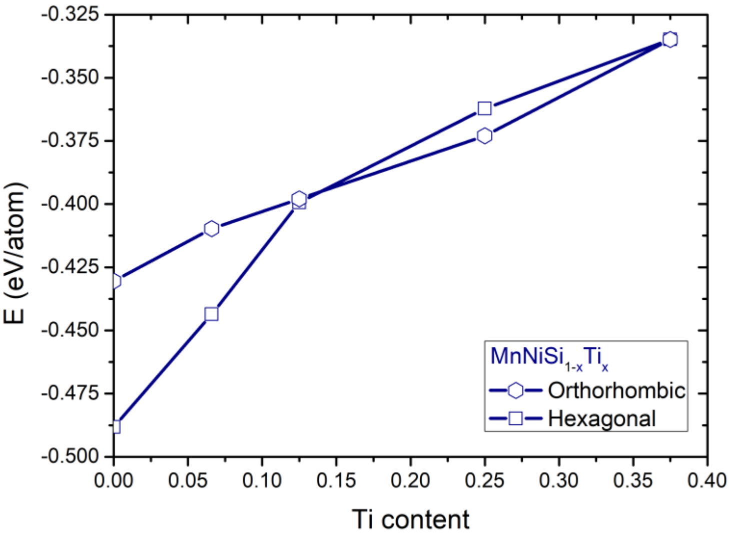

Figure S. 6: Isostructural substitution of Ti for Si in MnNiSi$_{1-x}$Ti$_x$. At around x=0.125 the hexagonal phase (hexagons) becomes more stable over the orthorhombic phase (squares).

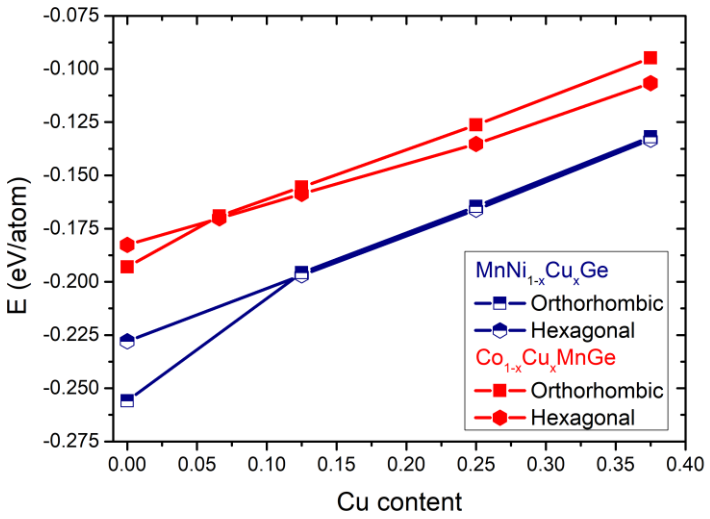

Figure S. 7 - Effects of Cu isostructural substitution in MnNi-CuGe (filled) and Co-CuMnGe (hald-filled) .The orthorhombic phase is represented by squares and the hexagonal by hexagons.
